# Source Space Estimation of Oscillatory Power and Brain Connectivity in Tinnitus

**DOI:** 10.1371/journal.pone.0120123

**Published:** 2015-03-23

**Authors:** Oliver Zobay, Alan R. Palmer, Deborah A. Hall, Magdalena Sereda, Peyman Adjamian

**Affiliations:** 1 MRC Institute of Hearing Research, University Park, Nottingham, United Kingdom; 2 National Institute for Health Research (NIHR) Nottingham Hearing Biomedical Research Unit, 113 The Ropewalk Nottingham, United Kingdom; 3 Otology and Hearing group, Division of Clinical Neuroscience, School of Medicine, University of Nottingham, Nottingham, United Kingdom; University of Regensburg, GERMANY

## Abstract

Tinnitus is the perception of an internally generated sound that is postulated to emerge as a result of structural and functional changes in the brain. However, the precise pathophysiology of tinnitus remains unknown. Llinas’ thalamocortical dysrhythmia model suggests that neural deafferentation due to hearing loss causes a dysregulation of coherent activity between thalamus and auditory cortex. This leads to a pathological coupling of theta and gamma oscillatory activity in the resting state, localised to the auditory cortex where normally alpha oscillations should occur. Numerous studies also suggest that tinnitus perception relies on the interplay between auditory and non-auditory brain areas. According to the Global Brain Model, a network of global fronto—parietal—cingulate areas is important in the generation and maintenance of the conscious perception of tinnitus. Thus, the distress experienced by many individuals with tinnitus is related to the top—down influence of this global network on auditory areas. In this magnetoencephalographic study, we compare resting-state oscillatory activity of tinnitus participants and normal-hearing controls to examine effects on spectral power as well as functional and effective connectivity. The analysis is based on beamformer source projection and an atlas-based region-of-interest approach. We find increased functional connectivity within the auditory cortices in the alpha band. A significant increase is also found for the effective connectivity from a global brain network to the auditory cortices in the alpha and beta bands. We do not find evidence of effects on spectral power. Overall, our results provide only limited support for the thalamocortical dysrhythmia and Global Brain models of tinnitus.

## Introduction

Subjective idiopathic tinnitus (TI) refers to a phantom sound that is consciously perceived without an external physical source. Several large population studies have suggested that the prevalence in adults is between 4.4 and 15.1% [1], and it is estimated that between 1 and 3 percent of the population are severely affected [2]. While these numbers clearly indicate the need for research into the condition, studies on TI also hold the prospect, on a more fundamental level, of yielding a better understanding of the workings of the central auditory system and the conscious perception of sounds [3].

**Fig 1:**
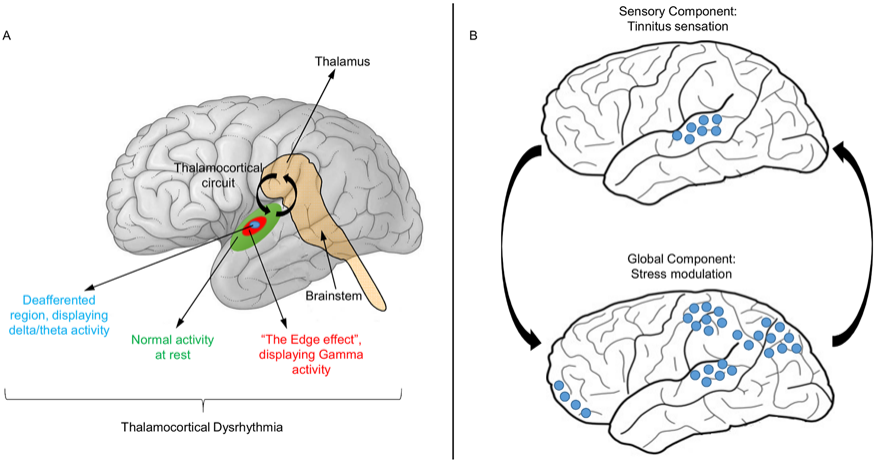
Neurophysiological models of tinnitus. (A) Thalamocortical dysrhythmia model [8,9]. In the deafferented region of the auditory cortex, thalamic inputs induce theta activity. Due to decreased lateral inhibition, this region is surrounded by an area of abnormal gamma activity (“edge effect”) leading to the TI percept. (B) Global Brain Model [13]. Decreased inhibition and increased excitability in the auditory cortices result from reduced sensory input. Tinnitus processing involves a globally extended fronto-parieto-cingulate network which amplifies the auditory neural activity by top-down influence. The level of tinnitus-related distress correlates with the extent of top-down amplification.

The pathophysiology of TI is complex and poorly understood. There is, however, some consensus between the neurophysiological models of TI regarding several key mechanisms of its pathogenesis. First, it is assumed that in many cases the root cause of subjective TI is altered input into the auditory pathway due to some damage to the peripheral hearing system [4–6]. Second, consequent changes in neural activity occur at different processing stages resulting in disinhibition and increased synchronicity of groups of neurons in the auditory cortex. Third, at the cortical level, these effects are thought to be specific to certain frequency bands of neuronal oscillation and should therefore present characteristic signatures in spectral analyses (for a review, see [7]). According to the influential thalamocortical dysrhythmia (TCD) model [8, 9], TI is the consequence of disrupted coherent activity between thalamus and cortex and correlates with gamma band activity (> 30 Hz). In the healthy brain, the thalamus fires at around 10 Hz in the resting awake state, thus driving the connected parts of the cortex to oscillate at the same frequency. This alpha activity is thought to be consequence of a mechanism of pulsed cortical inhibition [10–12]. During sleep, but also in the deafferented state, the thalamocortical neurons fire in the theta range (4–8 Hz). Thus, oscillatory abnormalities may arise from input deafferentation that causes overinhibition of thalamic neurons, which in turn reduces their excitatory drive. This is proposed to result in hyperpolarisation of membrane potentials of thalamic neurons and large-scale slow-wave activity that entrains the return thalamocortical pathways into theta oscillatory activity. At the cortical level, the focal slow-wave oscillations of cortico-cortical inhibitory interneurons reduce lateral inhibition and disinhibit gamma oscillations in neighbouring cortical regions. Consequently, abnormal gamma oscillations appear as an “edge effect” in neurons surrounding the theta-locked areas of the auditory cortex and form the neurophysiological correlate of the conscious TI percept (see Fig. 1A). Thus, according to this model, theta and gamma activity should arise from different but adjacent locations on the auditory cortex. Electroencephalography (EEG) and magnetoencephalography (MEG) do not possess sufficiently high spatial resolution to localise these areas but given their excellent temporal resolution, we are able to assess frequency-specific predictions of the model in more detail.

Building on the neurophysiological TI model of Jastreboff [4], the Global Brain Model (GBM) proposed by Schlee et al. [13] considers two main components, sensory and global, whose interplay results in the TI sound (Fig. 1B). The sensory component essentially comprises the auditory cortices and is again responsible for producing the neuronal gamma oscillations. These oscillations are thought to be due to a decreased alpha rhythm (8–12 Hz) caused by deafferentation and leading to gamma disinhibition. The global component consists of a distributed network of interconnected brain areas. It is hypothesized that this network is connected to the global workspace associated with consciousness, as postulated by Dehaene et al. [14]. Once the neuronal signal enters this workspace, the TI sound is perceived. Crucially, the global level also exerts a top-down influence on the sensory level. In particular, it strongly contributes to the decrease in alpha thereby perpetuating the perception of TI. The heuristic pathophysiological TI model proposed by de Ridder [15] is based on the TCD model, but describes a specific distress network whose activation brings about the negative emotional consequences of TI. Important nodes in this network are the amygdala, anterior cingulate cortex (ACC), anterior insula, parahippocampus, and the dorsolateral prefrontal cortex.

These various brain models make a number of predictions that can be tested non-invasively in humans with EEG or MEG. Most importantly, they predict characteristic changes in the power spectrum of the activity within the auditory cortices. The TI-related high-frequency oscillations should lead to a power increase in the gamma band. The TCD model also implies higher power in the theta band, whereas the GBM suggests a decrease in alpha power. A further consequence of the GBM is a negative correlation between alpha and gamma power [16].

A number of MEG and EEG studies have compared resting-state oscillatory activity in TI subjects to TI-free control groups, but so far a consistent picture has not emerged. Weisz et al. [17], [18] found increased power in the delta (1–4 Hz) and gamma bands of TI subjects and a reduction in alpha. This pattern was also observed by Adamchic et al. [19]. Although these results indicate slow-wave activity to be increased in the delta rather than the theta band, they can be considered as generally in agreement with the theoretical predictions. However, a number of studies reported different findings. Adjamian et al. [20] observed power increases in delta and theta, but could not detect significant differences for higher frequencies. Conversely, the results of Ashton et al. [21] showed differences only in the gamma band. Moazami-Goudarzi et al. [22] found increased spectral power for TI participants in the frequency range 2–100 Hz with significant differences in delta, theta and beta (12–30 Hz). Finally, the large-sample EEG study of Weiler and Brill [23] revealed a marked distinction between male and female TI patients with males displaying a *decrease* in power in delta, theta, alpha and beta (there defined as 14–21 Hz) compared to same-sex controls, and females an increase in theta, alpha, and beta (gamma was not considered). It is not clear whether the discrepancies between the studies are due to methodological differences, sampling error, the composition of the samples or other reasons, but they certainly warrant further investigation.

Connectivity analysis [24] provides a further opportunity for exploring the neural correlates of TI. It has been known for some time that the different brain areas do not work independently of each other, but are connected to each other in a variety of long-distance networks (see [25] for a review). This suggests that information processing and cognitive functioning are the result of interactions or communication between distributed brain systems. The basic purpose of connectivity analysis is to uncover relationships between different brain areas by detecting similarity or mutual influence between the corresponding source signals. One broadly distinguishes between functional and effective connectivity [26, 27]. Functional connectivity only assesses similarity (e.g. correlation) between signals. Effective connectivity also tries to establish causal relationships, i.e., to determine if one signal can be seen as the cause (or driver) of the other. It is expected that connectivity between areas varies between frequency bands, and connectivity measures should therefore be frequency-dependent [28].

The existing neurophysiological models predict TI to affect brain activity mainly in the theta, alpha and gamma bands, so it is plausible to regard connectivity changes in these frequency bands as supporting evidence for the TI models. The absence of connectivity changes in these frequency bands or changes in *other* regions should not be regarded as evidence *against* the models as the interplay between power and connectivity changes can be complex. Similarly, more detailed predictions, e.g., regarding specific pairs of nodes or the direction of the change in connectivity (i.e., increase or decrease), would require a solid understanding of the networks in which the auditory cortex is embedded and the various paths between the nodes. Such knowledge is not yet available.

A number of previous studies have investigated TI-related changes to brain networks. Following some initial observations by Shulman and Goldstein [29], the first evidence for the existence of a “tinnitus network” was given by Schlee et al. [30] using MEG. Comparing TI subjects and normal-hearing controls, they found abnormal functional connectivity in TI to be widely spread over the brain. In addition, connectivity between the ACC and the right frontal lobe as well as between the ACC and the right parietal lobe strongly correlated with TI intrusiveness. In a follow-up study, Schlee et al. [31] found an increase in functional connectivity for TI subjects in the alpha band and a decrease in gamma. They also showed that the gamma network changed with the duration of TI such that in participants with a TI duration of less than 4 years, the left temporal cortex was predominant in the gamma network, whereas in TI of longer duration, the gamma network was more widely distributed to include frontal and parietal regions. In yet another study by the same group, Schlee et al. [32] mapped cortical network hubs using an analysis of effective connectivity. The prefrontal cortex, the orbitofrontal cortex and the parieto-occipital region were among those areas whose connection strength was most strongly affected by TI compared to healthy controls. It was also found that the inflow into the temporal lobes significantly correlated with TI distress. For a review of related fMRI work, see [33].

In this study, we compare resting-state brain activity between TI participants and healthy controls, based on the analysis of MEG data. Given that a consistent and empirically confirmed picture of TI-related changes of cortical activity has not yet emerged, the motivation of this work is to provide further evidence useful for the assessment and refinement of neurophysiological theories of TI. More specifically, the first objective of our study is to perform a spectral analysis of the MEG data. Based on the theoretical TI models, we predict an increase of spectral power for participants with TI in the theta and gamma bands and a decrease in alpha. We expect that any such changes should be particularly pronounced in the auditory cortices. The second objective is to carry out a connectivity analysis in order to obtain further information about the TI network. The existing theories do not make specific predictions about connectivity changes. However, as any such effects must be related to TI-associated brain activity, we will consider alterations in brain connectivity in the theta, alpha and gamma bands as supporting evidence for the TI models, as discussed above.

Methodologically, the guiding principle for our analysis is the subdivision of the brain into sensory and global components put forward in the GBM [13]. Our primary analyses are carried out at the level of these components and comprise frequency-resolved comparisons between TI and control groups for sensory- and global-component spectra, as well as for connectivities within and between these components. Both functional and effective connectivity are considered in order to obtain complementary perspectives. To the best of our knowledge, such a combined study of the two types of connectivity has not been carried out so far in the context of TI. To implement the analyses described above, we develop an adaptation of the spatially hierarchical framework used by Hillebrand et al. [34], as discussed in more detail below. In a secondary, more exploratory analysis, connectivities are studied for a brain-wide network of localized regions of interest (ROIs). These ROIs underlie our modelling of the GBM as part of the hierarchical framework (see details of source analysis). These investigations provide a more detailed and fine-grained view of TI-related network changes; however, due to the much larger number of statistical comparisons, the evidence is not as strong as for the primary analyses. Further analysis is carried out to address the correlation between connectivity strength and a behavioural measure of TI handicap.

## Methods

### Participants

Participants with TI were recruited from the Nottingham Ear, Nose and Throat (ENT) clinic, Nottingham Audiology Services and NIHR Nottingham Hearing Biomedical Research Unit. Control subjects were found from the general population. TI subjects had chronic subjective TI for at least six months prior to recruitment. Exclusion criteria were pulsatile TI, Ménière’s disease, stapedectomy and neurological disorders. All participants were right-handed as assessed by the Edinburgh Handedness Inventory [35]. Ethical approval was granted by the Nottingham National Research Ethics Service (National Health Service) (code No. 08/H0408/89) and all participants gave written informed consent prior to enrolment. The cohorts included 28 TI participants (14 males, 14 females) with an average age of 54.7 years, and 19 non-TI controls (10 males, 9 females) with an average age of 39.0 years. The cohorts comprised the same participants as our earlier study [20], except for the addition of 5 controls and 2 TI subjects and the removal of 4 TI subjects based on the availability of data. The group of participants with hearing loss and no TI was not considered due to the low number of people (n = 8) in this group.

The severity of TI symptoms was assessed by the Tinnitus Handicap Inventory (THI) [36]. TI handicap was classified as slight (0–16), mild (18–36), moderate (38–56), severe (58–76), or catastrophic (78–100) [37]. According to these categories, 3 participants reported slight TI, 11 mild, 7 moderate, 5 severe and 2 catastrophic tinnitus. Hypersensitivity to sounds was assessed with a questionnaire where a score of > 28 is indicative of hyperacusis [38]. Based on this criterion, 3 participants in the TI group were classified with hyperacusis. Table 1 summarises the characteristics of the participants.

**Table 1:** Characteristics of participant groups.

Variable	Metric	TI	No TI
**Gender**	Male/Female	14/14	10/9
**Age**	Mean yrs (SD)	54.7 (12.8)	39.0 (14.0)
**PTA^1^**	Mean left (SD)	25.2 (18.5)	4.5 (6.4)
	Mean right (SD)	21.1 (16.5)	4.0 (5.7)
**THI^2^**	Mean score (SD)	39.9 (21.1)	NA
**Hyperacusis^3^**	Mean score (SD)	17.0 (9.4)	NA
	n^4^	3	NA
**TI Quality**	Tonal/Hissing/Ringing	19/3/6	NA
**TI Laterality**	Left/Right/Bilateral	10/6/12	NA
**TI duration**	Mean yrs (SD)	12.9 (15.1)	NA

^1^Pure-tone average (0.25–8 kHz).

^2^Tinnitus Handicap Inventory [36].

^3^Assessed by means of questionnaire [38].

^4^Subjects with hyperacusis (score > 28).

**Fig 2:**
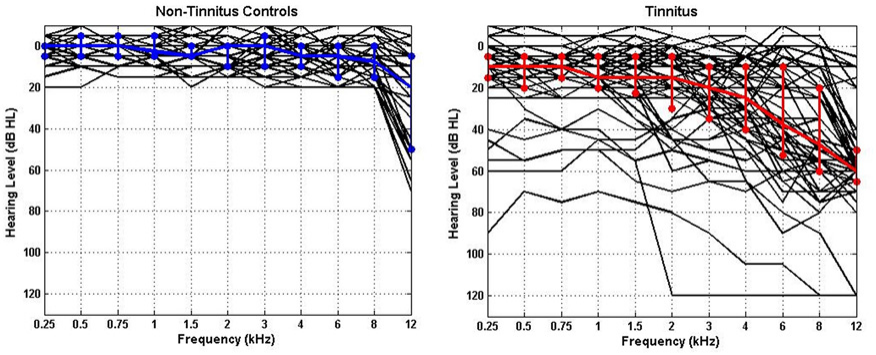
Individual (black lines) and median (coloured lines) audiograms for all ears in each group. (A) Non-TI controls (n = 19), (B) tinnitus participants (n = 28). The median thresholds are shown in blue and red, respectively.

The Tinnitus Tester software [39, 40] was used to measure TI laterality, loudness, dominant pitch, and quality. Further details of this procedure are provided in our previous papers [20, 41]. Pure-tone audiometry was collected for each participant for frequencies between 0.25 and 12 kHz. Individual audiograms and the median for each group are shown in Fig. 2, which illustrate the thresholds for all ears in each group. The variability in hearing thresholds for each group is also depicted using the 25% and 75% interquartile ranges. All non-TI controls had clinically normal hearing (i.e., thresholds ≤20 dB between 250 Hz and 8 kHz), but in most cases we found increased thresholds at 12 kHz indicating the existence of hearing loss (and deafferentation) at higher frequencies.

### MEG data collection

MEG recordings were obtained in a magnetically shielded room with a whole-head CTF system (VSM MedTech, Port Coquitlam, Canada), consisting of 275 radial gradiometers and an additional 29 reference gradiometers and magnetometers for ambient noise cancellation. Data was collected at a sampling rate of 600 Hz. Participants were lying in a supine position while three electromagnetic coils were attached to the nasion and left and right preauriculars for continuous head localization. Resting-state data consisting of alternating 1-minute segments of eyes-open and eyes-closed were obtained for a total of eight minutes. Participants were instructed to open or close their eyes through an earpiece. For each subject, MRI anatomical scans were obtained using a Philips 3T or 1.5T scanner, depending on availability. Images were T1-weighted magnetization prepared rapid gradient echo sequence, with a matrix size of 256x256x256 and a defined voxel size of 1×1×1 mm^3^. Co-registration with the MEG data was performed using a surface-matching technique [42].

### Data pre-processing

Raw MEG data were bandpass-filtered between 0.5 Hz and 48 Hz and downsampled to 150 Hz. The current analysis used the eyes-open periods of the resting-state data as they were considered to be less affected by artefacts after visual inspection. In order to exclude any transient effects, data segments of 5s and 1s length were removed from the beginning and end of the downsampled eyes-open periods, respectively. After applying the bandpass filter described above, the signal was found to still contain very strong line noise at 50 Hz. In addition, for many subjects the power spectrum also showed pronounced technical narrow-band noise around 21 Hz and 34 Hz (and sometimes at further frequencies). The origin of this noise is not known. To avoid confounding of connectivity estimates, the noise was removed using appropriately chosen notch filters (for further discussion of this approach, see Results section and S4 Appendix). All data analyses were performed with Matlab (The MathWorks, Inc., Natick, MA) and the FieldTrip MEG analysis package (http://fieldtrip.fcdonders.nl/start) [43]. Independent Component Analysis (ICA) was applied to remove artefacts such as heart beat and eye blinks [44].

### Source analysis

Source-space analyses are usually based on either a grid of voxels or a selection of ROIs. In this study, the ROI approach was chosen as results are more easily related to brain structure and theoretical TI models. We also expect this approach to have higher power for detecting TI-related effects.

**Fig 3:**
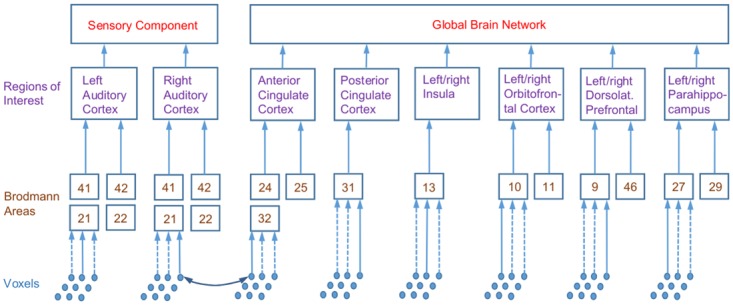
Spatial hierarchies used in source analysis. Diagram shows constituent Brodmann areas (BAs) of ROIs. Anterior and posterior cingulate cortex contain the listed BAs from both left and right hemisphere. Connectivity between two BAs is estimated by averaging over ten random pairs of voxels drawn from BAs, connectivity between ROIs and top-level component is obtained by averaging over respective lower levels. Abbreviations of ROIs used in the text: (l/r)AC: (left/right) auditory cortex, ACC: anterior cingulate cortex, DLPFC: dorsolateral prefrontal cortex, Ins: insula, OFC: orbitofrontal cortex, PCC: posterior cingulate cortex, PHC: parahippocampus.

One of the guiding principles of the current study was that analyses and computations are performed at various levels of a spatial hierarchy (see Fig. 3). The top level, which is used in the primary analysis, comprises the sensory and global components of the GBM. The second level of the spatial hierarchy consists of localized ROIs which are assumed to underlie the two components of the top level and which form the basis of our secondary analysis. More specifically, the sensory component is supposed to comprise the left and right auditory cortices (l/rAC). To increase power, the global brain network is modelled to consist of a limited number of ROIs which have been related to the TI network in previous work [15, 31, 45]. For anatomical identification, the ROIs are broken down into the constituent Brodmann areas (BAs), which form the third level of the spatial hierarchy.

The spatial hierarchy was motivated by the approach described by Hillebrand et al. [34] who studied brain networks at the level of Brodmann areas. This work computed connectivities between BAs by selecting a representative voxel for each BA, chosen as the grid point with the largest source power. In our framework, we follow a similar approach but add two further layers on top of the BAs, i.e., the localized ROIs comprising several BAs and the top-level sensory and global components (see Fig. 3). These additional averages reduce spatial specificity, but we expect to gain statistical power (as the number of comparisons is reduced) and increase the robustness of results.

To compute a quantity of interest at a given level of this hierarchy, we successively averaged over the underlying lower levels. For example, to compute functional connectivity between the top sensory and global component, we averaged over the connectivities between the underlying ROIs, which in turn are averages over the connectivities of the constituent BAs. To obtain the connectivity between two BAs, we randomly selected one voxel from each BA and computed connectivities between the voxels. This procedure was carried out ten times and BA connectivity estimates were obtained by averaging over repetitions.

Connectivities *within* the sensory component were computed by taking the mean over the connectivities within the left and right auditory cortices. These in turn were computed as averages over the connectivities between the respective constituent BAs. Connectivities within the global component were computed from connectivities between and within constituent ROIs (the latter for ROIs consisting of more than one BA).

To localize BAs in the individual subjects’ MEG space, the WFU pick atlas (Wake Forest University School of Medicine, Functional MRI Laboratory) [46] was used which contains an atlas of BAs for a Montreal Neurological Institute (MNI) template brain. In the WFU pick atlas, BAs were defined on a cubic grid with 2 mm spacing. Each hemisphere has 42 BAs, and the total number of grid points (voxels) assigned to BAs is 60299. To make the number of grid points more manageable, we increased grid spacing to 4 mm leaving 7619 points. For each subject, the MRI brain image was then spatially normalised to the MNI template brain using SPM8 (Wellcome Department of Imaging Neuroscience, UCL, London) and FieldTrip routines (i.e., a one-to-one transformation between points in the template and the subject headspace was established). Using this normalization and the MEG-MRI co-registration, the BAs were localized in each subject’s MEG space. Spatial group averages were then performed as averages over equivalent ROIs across subjects.

Source time series for virtual electrodes at the grid points defined above were computed using the linearly constrained minimum-variance (LCMV) vector beamformer with unit-noise-gain normalization [47–49] and the regularization parameter [50] set to 5% of the mean of the diagonal of the covariance matrix. Hillebrand et al. [34] provide a description of the technical details of this projection method together with a discussion of its adequacy for the purpose of connectivity analysis. Here, we only note that the LCMV beamformer requires the covariance matrix between sensor signals. In the present analysis, covariance matrices were computed separately for the four different intervals of eyes-open data for each subject.

### Spectral analysis

Power spectra were computed using the averaging scheme described above. For each BA, ten voxels were drawn at random. For each voxel, the source signal was split into segments of 2s duration which were Fourier transformed using the Hanning window. The power spectrum for the BA was obtained by averaging over the voxel spectra.

### Connectivity analysis

Functional connectivity was computed using the imaginary part of spectral coherency in order to suppress artefacts from source leakage [51]. Averages were computed from the absolute value of imaginary coherency to avoid problems resulting from the arbitrariness of signal polarity [34]. As a measure of effective connectivity, we employed partial directed coherence (PDC) [28]. The PDC value *π*_*ij*_ *(f)* of the connection from site *j* to *i* describes the strength of *j*’s influence on *i* relative to the total strength of *j*’s influence on all sites, including itself, at frequency *f*. For further details, see S1 Appendix.

### Analyses and statistics

Primary analyses were carried out on the level of the GBM and comprised comparisons between TI and control groups for the following measures: power spectral densities for the sensory and global component; functional connectivity (imaginary coherency) within the sensory component (i.e., within left and right AC), within the global component and between sensory and global component; effective connectivity (PDC) within sensory and global component as well as outflow from the sensory component to the global component and *vice versa*. In addition, correlations between these connectivities and TI handicap, as measured by the global THI score, were considered for the TI group.

All measures (spectra, connectivities, and correlations) depend continuously on frequency so that each of the comparisons actually presents a multiple-testing problem. To protect against type-I error inflation, we employed a cluster-based permutation test [52]: at each frequency, the two-sample independent t statistic for the comparison between TI and control groups was computed. Clusters were defined as contiguous regions along the frequency axis for which *|t|*≥2. The cluster weight equals the sum of the absolute *t* values within the cluster. The p value of the cluster was computed through a standard permutation procedure by randomly assigning subjects to the patient and control groups. For the correlations, the cluster inclusion criterion was an absolute value of individual correlations above 0.373. This is the critical limit in a standard significance test for a single correlation in a sample with the size of the TI group.

Formally, we consider each of the measures of the primary analysis as giving rise to a separate planned “family of comparisons” and provide multiple-comparison protection only within these families. Frequency regions containing significant differences between TI and controls are thus given by clusters with p values below 0.05. To provide further exploratory information, we also report clusters with p-values up to 0.1 as indicative of potential differences (note that p-values are not fixed absolute numbers but depend, e.g., on the overall width of the frequency interval examined). In the secondary analyses, connectivities and correlations were examined on the level of the localized ROIs. P-values were again computed for each comparison by means of a frequency-based permutation test. However, due to the vastly increased number of comparisons (e.g., connectivities for each pair of ROIs), the weight of the evidence is strongly reduced and the analyses are more of exploratory character.

Throughout the analyses, frequency is treated as a continuous variable. For descriptive purposes and more qualitative discussions, classical EEG frequency bands are assigned to frequency intervals of interest based on overlap: delta (1–4 Hz), theta (4–8 Hz), alpha (8–12 Hz), beta (12–30 Hz), gamma (>30 Hz).

## Results

### Primary analyses

#### Power spectra

**Fig 4:**
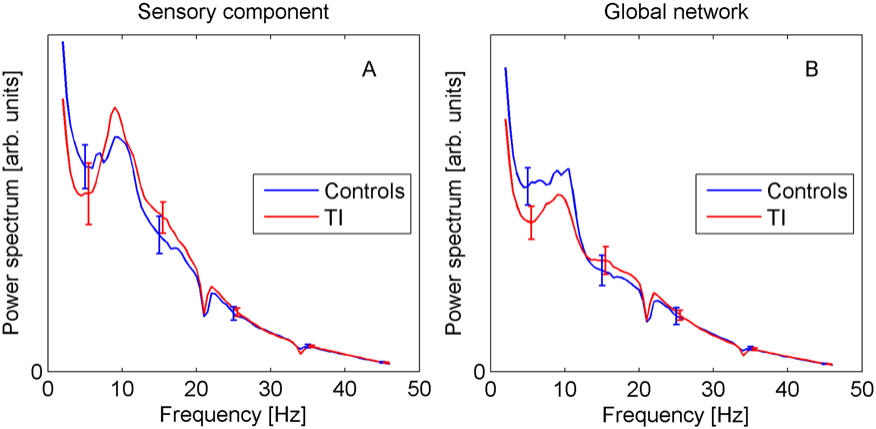
Power spectra for sensory (A) and global components (B). Error bars show standard error.

Based on the theoretical TI models, we predict an increase of spectral power for TI participants in the theta and gamma bands, and a decrease in alpha (see Introduction). Fig. 4 shows the group-averaged spectra for the sensory and global components. They display the expected 1/*f* decay with superimposed alpha peak. However, there is no statistical evidence for differences between TI and controls at any frequency (error bars in all plots show ±standard error). Note that the spectra display small dips around 21 Hz and 34 Hz, which are due to the notch filters applied to eliminate technical noise, as explained in the Methods section.

#### Functional connectivity (imaginary coherency)

Based on the theoretical TI models we expect changes in connectivity in the theta, alpha, and gamma bands, as TI-related activity should occur mainly in these frequency regions. However, a definite prediction remains difficult, and an absence of such changes, or changes in other frequency bands cannot be regarded as evidence against the models, as discussed in the Introduction.

**Fig 5:**
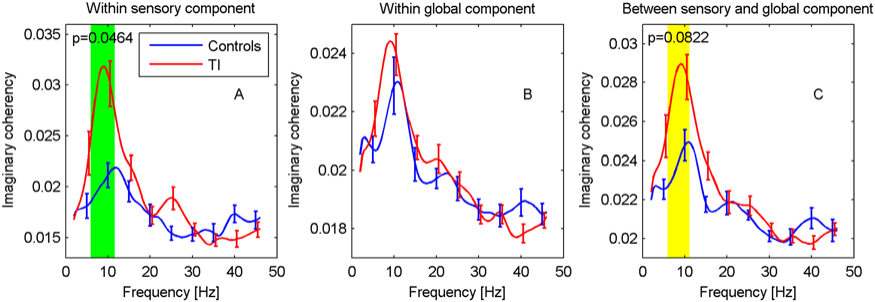
Functional connectivity for sensory and global components. Imaginary coherency is shown (A) within ACs, (B) within global network, (C) between ACs and global network. Frequency regions with significant differences (p<0.05, green) were determined with a cluster-based permutation test, regions with 0.05≤p≤0.1 are marked in yellow.

Results for imaginary coherency are shown in Fig. 5. Generally, the various curves display a peak around 10 Hz, which might perhaps be related to the alpha peak in spectral power. There is a statistically significant increase in functional connectivity for the TI participants within the auditory cortices in the frequency interval 6–11.5 Hz (p = 0.046). For a very similar frequency interval (6–11 Hz), there is an indication of a difference in connectivity between the auditory cortices and the global network (p = 0.082). No differences are found within the global network.

#### Effective connectivity (PDC)

**Fig 6:**
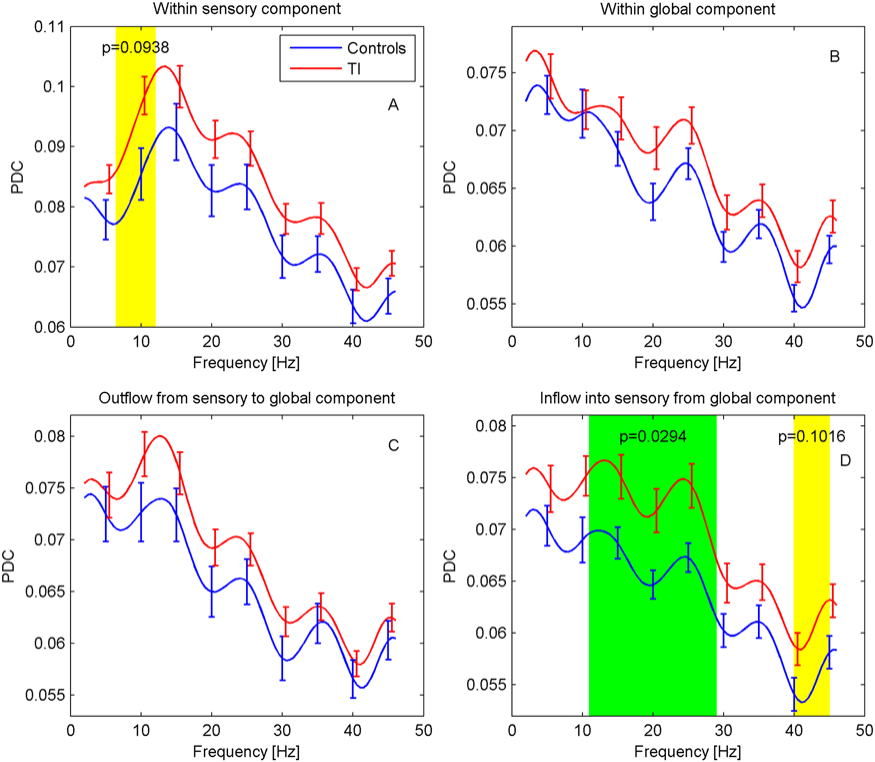
Effective connectivity for sensory and global components. PDC is shown (A) within ACs, (B) within the global network, and as (C) outflow from and (D) inflow into ACs from global network. Frequency regions with significant differences (p<0.05, green) were determined with a cluster-based permutation test, regions with 0.05≤p≤0.1 are marked in yellow.

For effective connectivity, we again expect any differences to predominantly occur in the theta, alpha, and gamma bands although more specific predictions are difficult to make. Results of the PDC analysis are displayed in Fig. 6. Overall, PDC shows a gradual decrease with frequency. For the PDC within the sensory component (ACs), a distinct peak around 12 Hz can be recognized. Connectivity for TI participants appears to be generally elevated. In particular, a significant difference is found in the inflow into the auditory cortices from the global network for the frequency interval 11–29 Hz (p = 0.029), together with an indication of a difference at 40–45 Hz (p = 0.102). The PDC within the auditory cortices also shows an indication of a difference in the interval 6.5–12 Hz (p = 0.094).

#### Correlations with TI handicap

**Fig 7:**
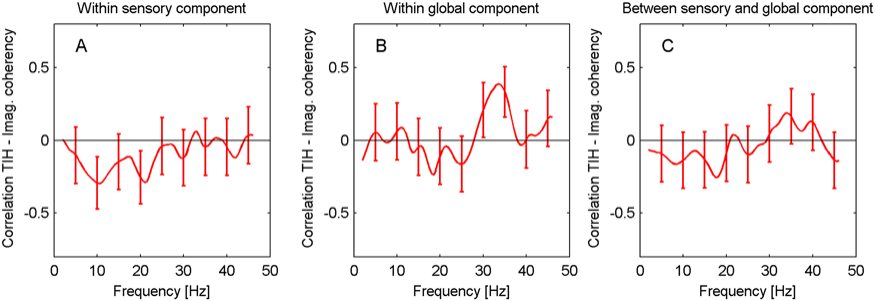
Correlations between TI handicap score and functional connectivity. Correlations for TI participants are shown (A) within the ACs, (B) within the global network, (C) between the ACs and the global network.

The theoretical TI models predict TI-related activity in the theta, alpha, and gamma bands. We therefore expect correlations between connectivities and behavioural measures of TI to predominantly arise in these frequency regions. Fig. 7 shows the correlations between the self-reported TI handicap (as measured by the global THI score, 0–100) and functional connectivity within ACs, within the global component, and between ACs and global component, respectively, for the TI group. In all cases, there are no significant correlations between THI scores and functional connectivity. The same result is found for the correlations between handicap and the different measures of effective connectivity (PDC) discussed above (correlation plots not shown).

### Secondary analyses

In the secondary analyses, spectra, connectivities and correlations are explored at the level of the ROIs (level 2 of spatial hierarchy, see Fig. 3).

#### Spectra

No significant differences between TI participants and controls were observed in the spectra for the individual ROIs. However, since there are many possible ways of statistically assessing the influence of TI on the power spectra, it is conceivable that other analysis approaches might be more sensitive and better suited to detect effects at the sample sizes of this study. With a view to inform future data analysis, we therefore carried out additional exploratory analyses following previously published methodology.

In a study involving subjects with a variety of neurological disorders related to thalamocortical dysrhythmia, Llinas et al. [9] computed the ratio of aggregate spectral power in the intervals 5–10 Hz and 10–15 Hz. According to the TCD model, one would expect this ratio to be increased in the patient group. For our auditory-cortex spectra, the mean ratio across all participants is 1.19 consistent with the overall decrease in spectral power with frequency, but there is no difference in ratios between TI and controls (means 1.18 and 1.20, p = 0.84 from permutation test).

Weisz et al. [17] performed a repeated-measures ANOVA with delta, theta and alpha power as levels of the within-subject factor and subject group as between-subject factor. For our AC data, we find neither a significant band x group interaction (*F*(1.47,66.1) = 1.764, p = 0.19 after Greenhouse-Geisser correction) nor a main group effect (*F*(1,45) = 0.175, p = 0.68). A further characteristic observation of Weisz et al. [17] that can be assessed even without recourse to the control group is a reduction of alpha power compared to delta in the TI group. For our data, we do not find such an effect in the auditory cortices (*t*(27) = -0.837, p = 0.41, in a paired t-test of delta-alpha, see also Fig. 4). Lorenz et al. [16] report pronounced negative correlations between alpha and gamma power in the *normalized* spectra. While we find a similar behaviour in our data, it remains unclear whether this observation describes a real biological effect. Rather, it could also be a mathematical artefact caused by the spectral normalization which unavoidably introduces correlations between the different frequencies. In the unnormalized data, the effect is not visible. Further work is necessary to clarify this question, but we note that evidence for the existence of negative alpha-gamma amplitude correlations was provided in a cross-frequency coupling study of macaque visual cortex [53].

#### Functional connectivity

Results of the ROI-based analysis of functional connectivity between the auditory cortices and the global network are displayed in S1 Fig. We find some node pairs with indications of differences (p<0.1) in frequency areas that more or less match the frequency intervals in the primary analysis (6–11 Hz) for which a similar difference was found (see Fig. 5). However, there are no nodes to which this difference can clearly be assigned. Its emergence thus appears to be the result of the aggregation over all ROIs in the global network. For the lAC-rOFC connectivity there is also a region in the gamma band that shows a significant difference (p = 0.030) although this region does not show up in the primary analysis. Within the global network, significant differences were found for the pairs rIns-PCC (39–44 Hz, p = 0.035) and rins-rDLPFC (36–40.5 Hz, p = 0.030).

#### Effective connectivity

**Fig 8:**
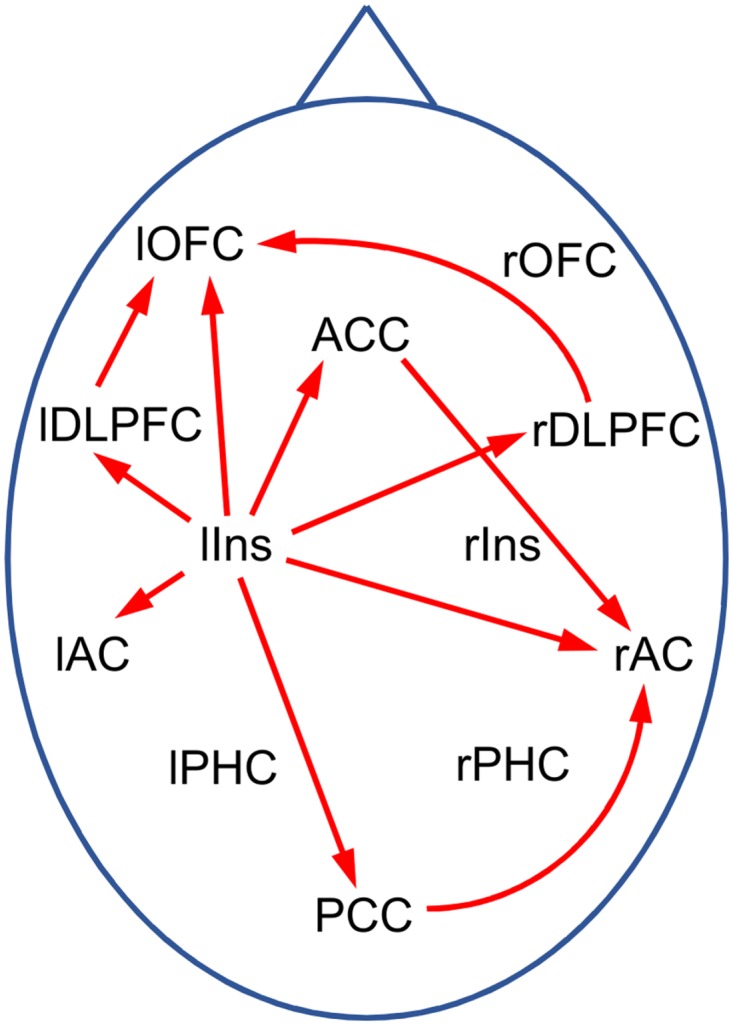
Tinnitus network as obtained from exploratory PDC analysis. Red arrows indicate connections with significant differences (p<0.05) between TI and controls in some frequency window. Abbreviations: AC: auditory cortex, ACC: anterior cingulate cortex, DLPFC: dorsolateral prefrontal cortex, Ins: insula, OFC: orbitofrontal cortex, PCC: posterior cingulate cortex, PHC: parahippocampus; l/r: left/right.

The primary analysis discovered significant differences in inflow into the ACs from the global network between TI participants and controls. It is thus of interest to further determine which ROIs in the global networks are responsible for these differences. A corresponding ROI-based analysis revealed the left insula as origin of the differential inflow into the left AC, and left insula, ACC and PCC as sources of inflow into the right AC (see S2 Fig.). As a further interesting result, it was found that the PDCs from the left insula to several target ROIs show significant differences between TI participants and controls (S3 Fig.) although no differences for PDCs within the global network as a whole were detected in the primary analyses. Altogether, the results of the secondary analyses for the effective connectivity suggest the “tinnitus network” shown in Fig. 8.

#### Correlations with TI handicap

A further analysis examined the correlations between the THI scores and individual functional and effective ROI-to-ROI connectivities. The results did not provide any evidence for an association, in agreement with the conclusions from the primary analysis.

### Additional analyses

In this subsection, we describe a number of additional analyses that were carried out to validate the results presented in Secs. 3.1 and 3.2. We investigate potential confounding effects of age, hearing loss, and noise contamination in the MEG signals, and compare the random-voxel-picking method used in the source analysis to alternative approaches.

### Age

The TI and control groups are not very well matched in age (54.7 vs. 39.0 years). This issue can be traced back to participant recruitment in our earlier study [20] which had groups ‘TI with hearing loss’ (n = 22, mean age 53.9 years), ‘TI with normal hearing’ (n = 8, 41.0 years), and ‘No TI with normal hearing’ (n = 14, 42.3 years). Pooling the two TI groups and deleting and adding several subjects (see Methods) leads to the differences in mean age described above.

Given the large difference in mean age, we investigated the existence of potential confounding effects with the help of an ANCOVA model that included group assignment (TI or control) as factor and age as covariate. For each of the primary spectral and connectivity measures, we assessed whether the model provided statistically significant evidence of an effect of age by means of a cluster-based permutation approach appropriate for linear models [54]. As no such evidence was found, we concluded that the effects of age are negligible in the present investigation. Details of these analyses are provided in S2 Appendix. However, to further investigate this issue, we have also related our findings to relevant results from other published studies. As explained in the Discussion section, also from this perspective it seems unlikely that the observed effects are due to the influence of age rather than the TI abnormality.

#### Hearing loss

In order to get an indication of whether the differences in hearing level between the control and TI groups might have a confounding influence on our results, we repeated the analyses of our primary outcome measures including the average binaural hearing level between 0.25 and 4 kHz as a covariate. The calculations were carried out analogously to the analyses for age described above. Again, we did not find any evidence that the conclusions of our original analysis are affected by the differences in hearing level between the control and TI groups. A more detailed description of the analysis is given in S3 Appendix.

#### Noise contamination

As noted in earlier, for a number of subjects the sensor signals were found to be contaminated with narrow-band noise at 21 and 34 Hz and sometimes other frequencies. In order to assess the effect of this noise on the analysis results, we compared the computations of the primary outcome measures with and without applying the corresponding band-stop filters. It was found that the differences in the group averages as well as in the observed t-statistics were minimal (see S4 Appendix). This observation indicates that the narrow-band noise does not have any substantial influence on the analysis and conclusions and does not present a serious confounding effect. Also note that the beamformer analysis used in our study has been shown to be a robust technique for removing pronounced noise in the sensor data [55].

#### Validation of random-voxel-picking approach

To compute the connectivity between two BAs, one would ideally average over the connectivities of all pairs of constituent voxels. However, due to the large number of voxels making up some of the BAs such calculations are too time consuming to be practically feasible. To reduce computation time, it would therefore be useful to base calculations on a small number of representative source signals for each BA. Hillebrand et al. [34] choose the voxel with the largest source power to assign a time series to a BA, as described above. However, there is no compelling reason to use this particular selection method and Hillebrand et al. [34] provide alternative choices. In the present paper, a voxel is selected *randomly* from each BA, and connectivities are computed from this random set of voxels. After carrying out this procedure ten times, BA connectivity estimates are obtained by averaging. As this procedure is not described in Hillebrand et al. [34], we validated it by systematically comparing our functional connectivity results to computations based on two other methods. In the first method, each BA is represented by the voxel whose signal correlates most strongly with the signals of all other voxels within the BA, as suggested in [34]. For the other approach, we selected 20 voxels in each BA that are spread out evenly across the constituent grid points. For each pair of BAs, the functional connectivity was determined as the average over the connectivities for all 20x20 pairs of voxels. Compared to the original method, we expect this approach to be more accurate as it is based on 20 voxels selected to be spread out across the BA rather than 10 randomly drawn ones, and it uses 20x20 = 400 connectivities rather than 10 (each random voxel in one BA is paired with one from the other BA).

We find that the results from both approaches are in good agreement with the random-voxel calculations, and we thus conclude that our results are reliable and not unduly affected by artefacts of voxel selection. In addition, we also found very good agreement between our results for the spectra and calculations using all voxels in each BA (for details of these analyses, see S5 Appendix). Altogether, the random voxel-picking approach appears to present a good compromise between assigning a single time series to each BA, which may not completely capture the signal variation within the BA, and methods using a large set of time series per BA which may yield very robust results but carry a high computational cost.

## Discussion

The main objective of our investigation was to search for neural correlates of TI by analysing MEG resting-state data obtained from 28 participants with TI and 19 controls and to compare our findings to the TCD and global brain models of TI. The analysis of power spectra did not provide any evidence of significant differences between TI and controls. Functional connectivity was increased for TI within the sensory component (auditory cortices) in the alpha band. A significant increase was also found for the effective connectivity between the ACs and the global network in the alpha and beta band. There was no evidence of correlations between connectivities and the score on THI.

### Hearing loss

As stated above, the purpose of the present work was to compare spectral and connectivity measures between participants with TI and non-TI controls. Given the recent findings indicating that the clinical audiogram is an insufficient measure of cochlear integrity [56], in the current study we did not—in contrast to our previous work [20]—distinguish between TI with and without hearing loss at clinical frequencies and rather pooled the corresponding subjects in the TI group. Combining these participants is justified by the conclusion of this work that a distinction between TI with and without HL is not meaningful since TI is likely associated with some degree of damage to the auditory system. Importantly, pooling TI participants increases the power of the statistical analyses.

We did not include the group of subjects with hearing loss and no TI from our previous work [20] in the current analysis as we considered the sample size (n = 8) to be too small. As a consequence, the present study does not allow to separate out the effects of hearing loss and TI.

### Power spectra

As described in the Introduction, the current literature does not provide a clear and unambiguous empirical picture of how TI affects resting-state spectral power. Different patterns of spectral changes have been reported, and observations are not replicated between studies. At the moment the theoretical TI models are neither confirmed nor ruled out.

The present analyses did not find any statistically significant differences in the power spectrum. Generally, power is expected to decrease with age [45]. Given the difference in mean age between the TI and control groups, we therefore cannot exclude that the lack of evidence for the expected power increase in the delta/theta and gamma bands is due to an age effect (even though the analyses did not find evidence of age effects in the present study). However, the absence of evidence for an alpha power decrease cannot be explained in this way.

The lack of evidence for spectral power differences should not be taken to mean that such effects do not exist, in principle. Rather, based on the theoretical work it still seems reasonable to expect that TI has a measurable effect on cortical power spectra. Providing unambiguous empirical evidence for this effect remains an important stepping-stone towards a better understanding of the neural mechanisms of TI. However, taken together with the previous inconclusive results of the literature, the current findings suggest that the corresponding effect sizes may be quite small. Effect sizes are determined by both the mean TI-induced actual change in spectral power and the variability within the TI population. It seems likely that the latter also considerably contributes to the problem. Recent connectivity studies have demonstrated heterogeneity between TI subpopulations, e.g., short- and long-duration TI [31], or early and late onset [45]. To observe TI-related spectral changes more clearly, it thus might be advisable to work with larger sample sizes and focus on suitably defined subpopulations, in addition to standardization of data collection and analysis protocols to facilitate comparison and meta-analysis of studies.

We note that in our earlier study [20], we found an increase in spectral power in the delta band which is not replicated here. We can envisage several reasons that could explain this discrepancy. First, the subject groups do not coincide with the previous study as in the current analysis there are more controls and some changes in the TI group (see Methods). Second, there are variations in the analysis methodology, in particular regarding the selection of voxels. Third, the raw datasets used in the analyses are not the same, and the paradigm (resting-state versus silence-masker) is very different. Finally, it is also possible that the variation is simply due to statistical fluctuations.

### Functional connectivity

Our analysis revealed a significant difference in functional connectivity within the auditory cortices for the theta and alpha bands (frequency interval 6–11.5 Hz). The general possibility of connectivity differences within the ACs is consistent with current theoretical neurophysiological models of TI. For example, the heuristic pathophysiological model of de Ridder [15] envisages that TI can be caused by hyperactivity in either the lemniscal pathway which projects to the primary AC or the extralemniscal pathway which projects to the secondary auditory cortex. Such a change of activity in one subregion of the AC could then affect connectivity to the other parts thus leading to the differences observed in our analysis.

In addition to the difference in connectivity within the ACs, we also find an indication of an increase in connectivity between the AC and the global network in a similar frequency region. Secondary analyses suggest that the difference cannot be tied to specific ROIs in the global network, but rather appears to emerge as an aggregate effect across all global-component ROIs.

Our results show an increase in functional connectivity for the TI group. A comparison of two groups of healthy controls with a difference in mean age of 30 years which was part of the EEG study [45] did not find differences in functional connectivity except for a decrease with age for two pairs of nodes in the gamma band. On the basis of this observation and our ANCOVA analysis, it seems unlikely that the connectivity increase reported here is due to an age effect.

The GBM of Schlee et al. [13] predicts a decrease in alpha activity in the auditory cortices. Our finding of a significant change in functional connectivity within the ACs in the alpha band thus provides support for this model. (Note that due to the complexity of brain dynamics a decrease in activity can be accompanied by a decrease but, alternatively, also an increase in connectivity. In the absence of more detailed hypotheses, our observation of an increase is thus consistent with the GBM.) A further study by Schlee et al. [31] found a change in functional connectivity in the alpha band as well; however, there a decrease was reported whereas our study observed an increase. As [31] considered whole-brain connectivity the two findings cannot be compared directly, but, nevertheless, it also is not clear whether they are compatible. A further difference is that these authors used the phase-locking value (PLV) [57] as functional connectivity measure which is very similar to absolute coherency. However, the PLV is vulnerable to confounding by source leakage [34].

Schlee et al. [31] also find a negative correlation between alpha and gamma connectivity. In contrast to the corresponding result for the spectra, it was ascertained that this observation is not an artefact from normalization. After normalizing the imaginary coherency at the level of the BAs, we also find negative correlations between connectivities in the alpha (8–12 Hz) and gamma band (40–46 Hz, the upper limit of our analysis). At the top level of our hierarchy and combining all subjects, these correlations range from -0.52 (connectivities between ACs and global component) to -0.59 (within ACs). However, before normalization these correlations range between -0.10 and -0.15.

Since our results show an increase in theta connectivity they provide some supporting evidence for the TCD model. However, the primary prediction of this model is an increase in theta activity (spectral power) which is absent in our data. The picture is thus not clear-cut. As confirmed by further analysis the observed connectivity increase is caused by an increase in covariance (synchronicity) between the nodes in the absence of any significant change in the average spectral power. It is not clear how this observation relates to the theoretical models of TI.

Note that our study did not consider connectivity between the ACs. This is due the fact that the TCD and global brain model describe a more hierarchical mechanism involving thalamus, ACs, and the global network in which effects on the between-AC connectivity are not given prominence. We also did not distinguish between left- and right-ear TI and thus do not stress differences between hemispheres in the connectivity index. This is because previous studies are not clear about the laterality of TI and which hemisphere should reflect the abnormalities. Moreover, in order to distinguish between hemispheric effects of TI, sufficiently large numbers of unilateral (left and right) and bilateral TI participants are needed to achieve adequate statistical power. This is an important consideration for future studies.

### Effective connectivity

The primary analysis of effective connectivity shows a significant increase of inflow into the ACs in the high-frequency alpha band and the beta band. In their MEG study of age-related changes in effective connectivity, Schlee et al. [58] do not find any evidence of age effects on the inflow into the ACs so that it appears unlikely that our observation is due to a confounding by age.

Fig. 6 suggests a general increase in PDC in the TI group, although the statistical analysis only found two further regions with indications of differences between TI and controls, i.e., in the gamma band for inflow into the ACs, and in the alpha band for PDC within the ACs. At present, it is not clear whether this general increase could be confirmed in a study with larger power or whether it is a sampling effect. The significant increase in inflow into the ACs could be interpreted as an indication of the top-down influence of the global onto the sensory component postulated by the GBM, but it is not obvious how an overall elevation of PDC in the TI group might be explained by this model. Leaving the framework of this model, however, it is conceivable that one contribution to the increase in PDC originates from differences in how tinnitus subjects and controls focus their voluntary attention. As TI participants are aware of their participation in a tinnitus study, they are likely to focus their attention on it their tinnitus, whereas controls might direct their attention in a variety of ways. Our design did not attempt to control for effects of this kind. Behavioural studies have suggested a link between tinnitus and impaired attention [59, 60], while brain imaging studies have revealed the involvement of various non-auditory structures in tinnitus that are linked to cognitive processes [61](Lockwood et al., 2001). These findings indicate that attention maybe necessary for conscious awareness of tinnitus, or task involvement may suppress the abnormal tinnitus-related activity [62]. Future studies should attempt to delineate the effect of attention at local auditory cortex and at the network level.

The secondary analyses indicate that the left insula as well as ACC and PCC are the main sources of the increased inflow into the ACs. More specifically, S2 Fig. suggests that in the tinnitus brain the ACC and PCC have an increased influence especially on the rAC, whereas the increased inflow from the left insula significantly affects both ACs, but more strongly lAC. Furthermore, Fig. 8 suggests the existence of a TI network in which the left insula plays a central role. Schlee et al. [32] conducted a grid-based analysis of effective connectivity in TI participants. In particular, they found one cluster in the orbitofrontal cortex with significantly increased inflow, and two clusters around the PCC and the cerebellum with reduced inflow, but no significant changes in inflow around the ACs. In addition, they found several regions with changes in outflow, but none of them involved the left insula. While our findings thus do not confirm the results of Schlee et al. [32], they are also not in obvious contradiction due to the differences in the analysis methods.

Previous studies have shown the insula to be involved in the TI network [45, 63–65], but in none of these studies does the insula seem to play as central a role as in the current analysis. We therefore cannot exclude the possibility that our observation is biased by statistical fluctuations. However, insular activity is related to feelings of distress [66, 67] so that the inclusion in the TI network is not surprising.

The results of our functional and effective connectivity analyses are quite different. While we find a significant difference in effective connectivity between ACs and the global component in the beta band such an effect is absent in the functional connectivity analysis. The latter also fails to find a significant change in connectivity for the left insula. In order to assess whether our mathematical analyses are consistent we have computed the imaginary coherency from the MVAR fit to the MEG data. The MVAR fit forms the basis of the PDC calculation, but can also be used to compute other types of connectivities [68]. As we find good agreement between the MVAR coherency and the nonparametric calculation of Fig. 5 we conclude that the MVAR model provides an appropriate description of the data and thus that the PDC calculations are not in any contradiction with the results from the imaginary coherency. The difference between the results for coherency and PDC thus highlights the fact that these measures provide independent views of the general concept of connectivity which cannot easily be related to each other. In view of the above considerations, the observation of significant differences in the beta band should thus not be seen as a direct contradiction to the theoretical TI models.

### Association with TI Handicap

Our primary analyses do not show any evidence of correlations between the TI handicap score and the connectivities related to the ACs and the global component. This observation is confirmed by our secondary analyses which consider connectivities for the individual ROIs. Correlations between behavioural TI measures and connectivities have previously been observed in a number of studies. Considering TI distress as measured by a German tinnitus questionnaire [69], Schlee et al. [30] found significant correlations with the PLV between the ACC and a right parietal source, as well as with the coupling between the PCC and a right frontal source. The PDC analysis [32] detected correlations between this distress measure and the inflows into clusters in the temporal cortices. The fMRI study of Ueyama et al. [70] identified a number of regions, e.g., in the cingulate cortex, whose aggregate connectivities correlated with THI. Given the results of these and other studies [65] which all had sample sizes similar to ours, we expected to also find clear evidence of correlations with THI in our data. The lack of significant correlations (see Fig. 7) which would persist even without multiple-comparison corrections (i.e., all individual correlations are below 0.373) is thus not easily reconciled with the existing literature.

### Conclusions

Overall, our results are not in contradiction with the theoretical TCD and Global Brain models of TI, but they also provide only very limited support. We find a significant increase in functional connectivity for TI in the theta and alpha bands. The TCD and global brain models of TI predict the theta, alpha and gamma oscillations to play a particular role in the generation of TI, so this observation provides some supporting evidence for these models.

As to the analysis of partial directed coherence, we find a significant increase of inflow into the auditory cortices in the alpha and beta bands. Although the differences do not reach significance anywhere else, the analysis results also raise the impression of a generally elevated PDC score for participants with TI. PDC is calculated via a complicated mathematical modelling of the observed neuronal signals, and it is difficult to relate the numerical results to the simple theoretical TI models (see, e.g., the comments above on the comparison between the PDC and coherency results from MVAR modelling). In this sense, our findings do not provide a clear contradiction of the theoretical predictions, but due to the distribution across frequency bands they also do not present strong support. In all other cases, in particular for the spectra, association with TI handicap score, and any gamma-related effects, we do not observe any significant differences. These findings should not be interpreted as contradictory to the theoretical predictions (in the sense of “evidence of absence” of effects), but rather as an absence of evidence which does not allow further conclusions to be drawn.

We acknowledge that the imperfect matching of our subject groups with respect to age and hearing level represents a potential limitation of our study. To assess the extent of possible confounding effects, we have conducted auxiliary statistical analyses and related our findings to previous results in the literature. Even though these arguments cannot provide certain proof, they seem to make it plausible that our main conclusions are not severely distorted by confounding.

In spite of the inconclusive results, we believe that our study makes a valuable contribution to the ongoing research into the neuronal origins of TI. A particular problem for testing the theoretical TI models is that they do not provide any quantitative predictions about effect sizes. In the absence of a solid basis for power analysis, it thus appears reasonable to start by conducting studies of moderate size such as the present one. However, the present results as well as the inconsistencies between the results of various similar recent studies suggest that sample sizes need to be increased substantially to obtain clear evidence of effects. In addition, it might be helpful to focus on particular well-defined TI subpopulations in order to reduce variability, and to standardize data analysis protocols to facilitate comparison and meta-analysis [33].

Another possibility for further research is to search for alternative electrophysiological markers that might be more sensitive to TI-related effects. Finally, we mention that our analyses point to a special role of the left insula in the TI network. This observation might also warrant further investigation. Overall, the identification of the neural correlates of TI remains as an important question which remains unresolved and it is hoped that the current results will be useful for informing the design of future, potentially larger, studies.

## Supporting Information

S1 Fig Functional connectivity between left/right AC and ROIs in global brain network. Frequency regions with significant differences (p<0.05, green) were determined with a cluster-based permutation test, regions with 0.05≤p≤0.1 are marked in yellow. Controls are shown in blue and TI subjects in red.
(TIFF)

S2 Fig Effective connectivity from ROIs in global brain network to left and right AC. Controls are shown in blue and TI subjects in red.
(TIFF)

S3 Fig Effective connectivity from left insula to all other ROIs. Controls are shown in blue and TI subjects in red.
(TIFF)

S1 Appendix Computation of connectivities. (DOC)

S2 Appendix Analysis of age effects. (DOCX)

S3 Appendix Analysis of effects of hearing loss. (DOCX)

S4 Appendix Analysis of effects of noise contaminations. (DOCX)

S5 Appendix Validation of random-voxel-picking approach. (DOCX)
