# Supplementary figures and images for: Source Space Estimation of Oscillatory Power and Brain Connectivity in Tinnitus
Source: PLoS One. 2015 Mar 23;10(3):e0120123. doi: 10.1371/journal.pone.0120123 (PMC4370720; doi:10.1371/journal.pone.0120123)

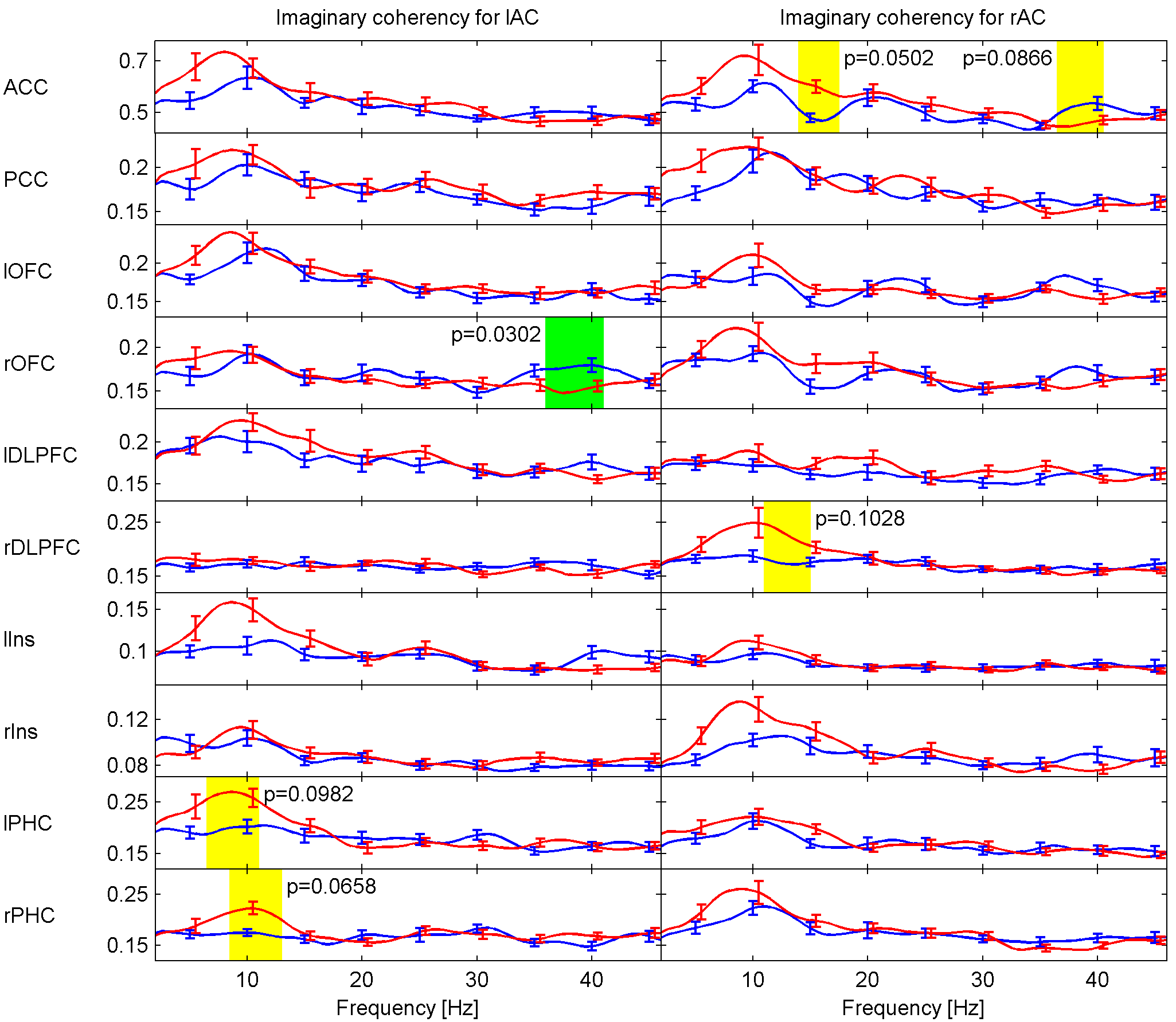

Supplement: S1 Fig — Frequency regions with significant differences (p<0.05, green) were determined with a cluster-based permutation test, regions with 0.05≤p≤0.1 are marked in yellow. Controls are shown in blue and TI subjects in red. (TIFF) [file pone.0120123.s001.tiff]

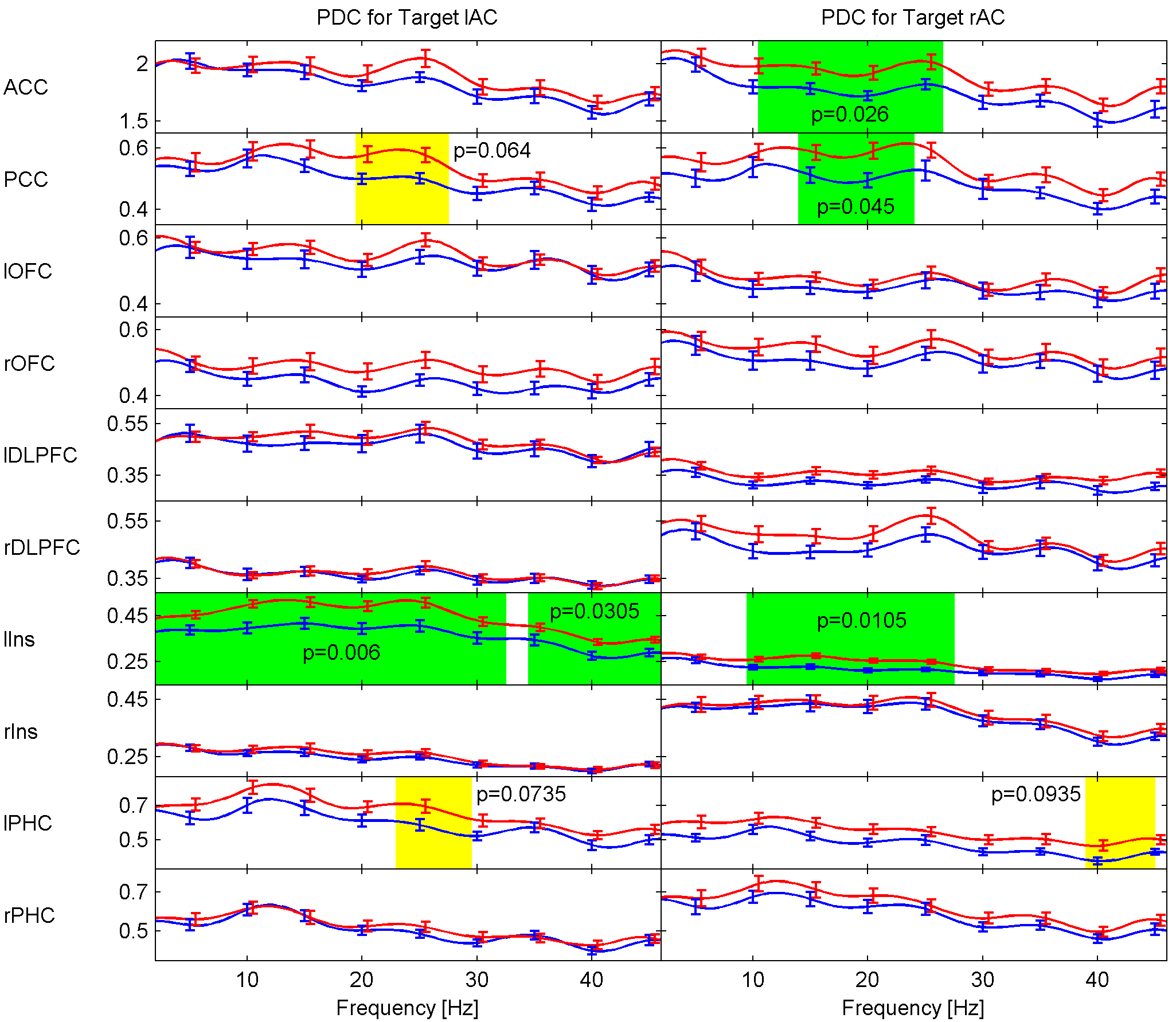

Supplement: S2 Fig — Controls are shown in blue and TI subjects in red. (TIFF) [file pone.0120123.s002.tiff]

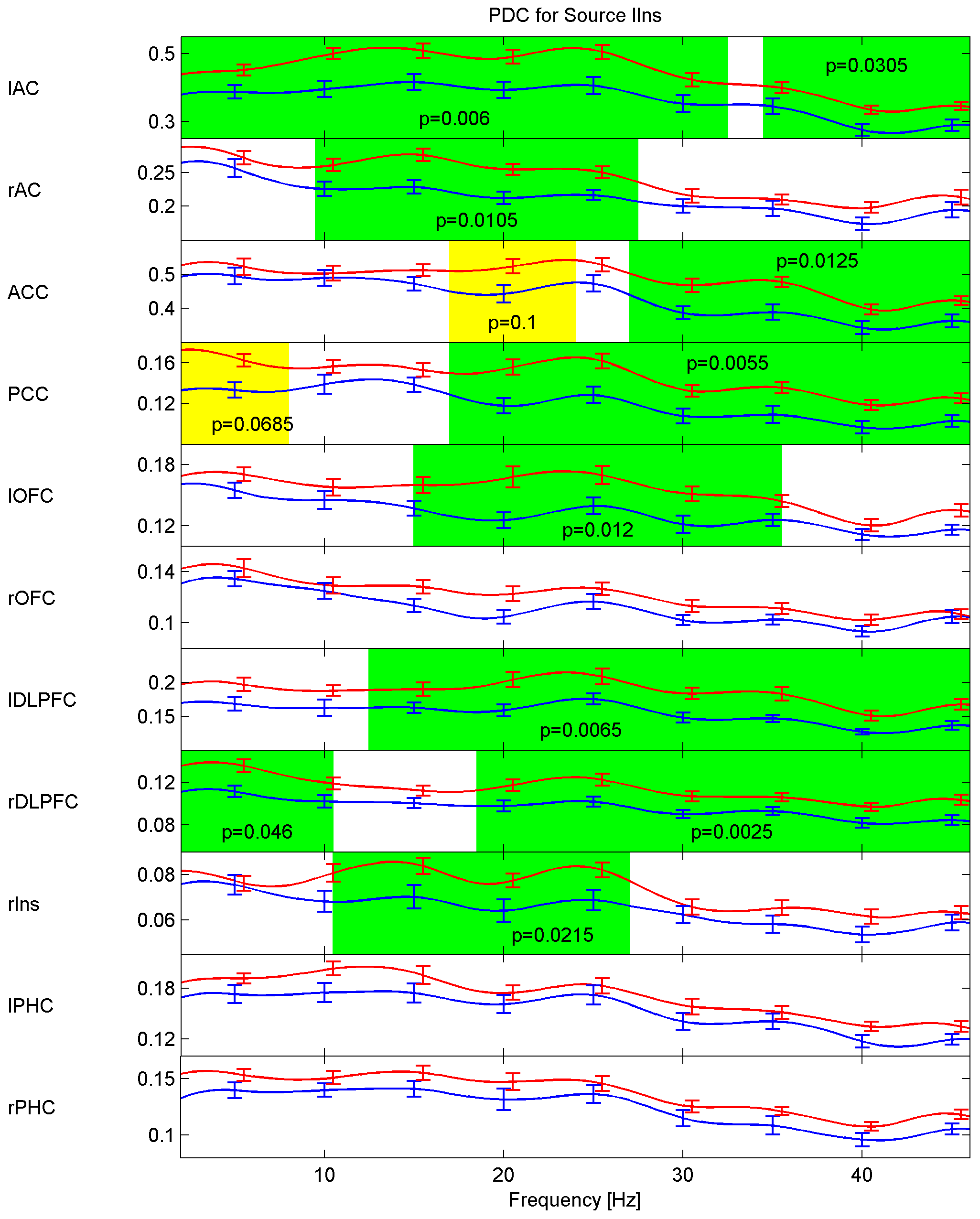

Supplement: S3 Fig — Controls are shown in blue and TI subjects in red. (TIFF) [file pone.0120123.s003.tiff]
