# Supplementary material for: Source Space Estimation of Oscillatory Power and Brain Connectivity in Tinnitus
Source: PLoS One. 2015 Mar 23;10(3):e0120123. doi: 10.1371/journal.pone.0120123 (PMC4370720; doi:10.1371/journal.pone.0120123)
Supplement: S1 Appendix — (DOC) [file pone.0120123.s004.doc]

Appendix: Computation of connectivities

We use the imaginary part of spectral coherency to measure functional connectivity. Spectral coherency between locations *i* and *j* at frequency *f* is defined as *C_ij_(f)= S_ij_(f)/√(S_ii_(f)*S_jj_(f))* with *S_ij_(f)* the complex cross-spectral density and *S_ii_(f*) and *S_jj_(f)* the power spectral densities . It can thus be considered as a kind of frequency-resolved complex correlation between the signals at *i* and *j*. As discussed in detail by Nolte et al. , it is advantageous to consider the imaginary part of *C_ij_* in order to suppress artefacts from source leakage. However, one has to be aware that this also removes any true zero-lag connectivity. In the present analysis, the spectral coherency *C_ij_(f)* was computed from data segments of 2s duration and the Fourier transform was carried out using the Hanning window, as for the spectral analysis (see Section 2.5).

To compute effective connectivities, we randomly draw a voxel from each BA in our model, fit a multivariate autoregressive system of lag 12 to this set of voxels and compute PDCs for each voxel pair. This procedure is again carried out ten times to obtain PDCs between BAs by averaging. To describe the total strength of *j*’s influence on all other sites (outflow), Schlee et al. (2009b) summed up all PDC values for source *j*, i.e., *Σ_i≠j_ π_ij_(f).* Similarly, in order to measure how strongly site *i* is influenced by the other sites (inflow), all PDC values with target *i* were added together. For comparability with Schlee et al. , these outflow and inflow measures will be used in the present work. For example, the inflow into an ROI is computed by summing over the PDCs from all other ROIs. These in turn are obtained by averaging over PDCs between constituent BAs.
