# Supplementary material for: Source Space Estimation of Oscillatory Power and Brain Connectivity in Tinnitus
Source: PLoS One. 2015 Mar 23;10(3):e0120123. doi: 10.1371/journal.pone.0120123 (PMC4370720; doi:10.1371/journal.pone.0120123)
Supplement: S2 Appendix — (DOCX) [file pone.0120123.s005.docx]

**Appendix S2 - Confounding effects of age**

There is a substantial age difference between the control and TI groups of more than 10 years. We have investigated potential confounding effects of age with the help of an ANCOVA model that includes age as a covariate. For each of the primary spectral and connectivity outcome measures it was assessed whether there is evidence of a statistically significant effect of age. If there is no such evidence, then the Age covariate can be discarded from the further analysis. This approach is very similar in spirit to the treatment of age in [[1](#_ENREF_1)].

***Method:***

The ANCOVA model under study reads y = β_0_+β_1_*Group+β_2_*Age+ε with y one of the MEG outcome measures (e.g., global or sensory-component spectral power, functional and effective connectivity) at frequency f and Group an indicator variable for group membership (tinnitus/control). For a single frequency f, it would be straightforward to test for significance of the Age coefficient β_2_; however, we have to simultaneously test at a sequence of frequencies and are thus faced with a multiple-comparison problem. In accordance with the statistical analyses in the main paper, we applied a cluster-based permutation test to deal with the multiple comparisons. Since the current model is more complex than the original one of the manuscript (which would amount to y = β_0_+β_1_*Group), the testing framework has to be adapted accordingly. Our method is based on Ref. [[2](#_ENREF_2)] and proceeds as follows.

The statistic used in permutation testing of β_2_ is the partial correlation between the MEG outcome measure y and Age, controlling for Group. The permutation distribution of the statistic under the null is computed based on permuting the residuals of the regression of y against Group and recomputing the partial correlation as described in [[2](#_ENREF_2)] (method of Freedman and Lane [[3](#_ENREF_3)]). The cut-off criterion for cluster detection was chosen as |part. corr.| > 0.287. This corresponds to the critical limit in a standard significance test for a single partial correlation in a sample with the size of the combined control and TI groups.

***Results:***

The following diagrams show the partial correlation as a function of frequency for the various outcome measures. The cluster cutoff criterion is indicated by the horizontal lines. Clusters are determined by the segments of the curve above the upper or below the lower horizontal lines, respectively. The p-values of these clusters are determined based on the permutation test.

*Spectra:*

Sensory component Global component


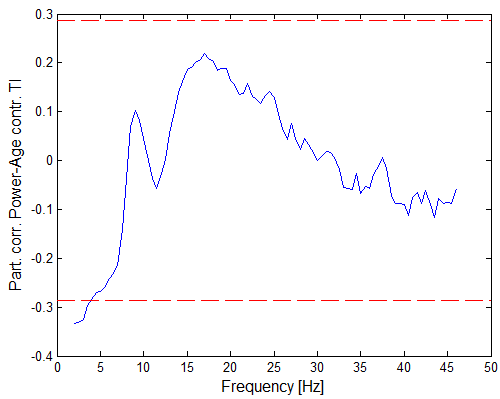

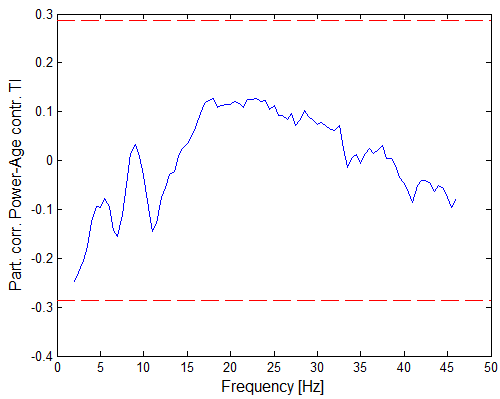


Cluster p-value: 0.19 No clusters

*Functional connectivity:*

Within sensory component Within global component


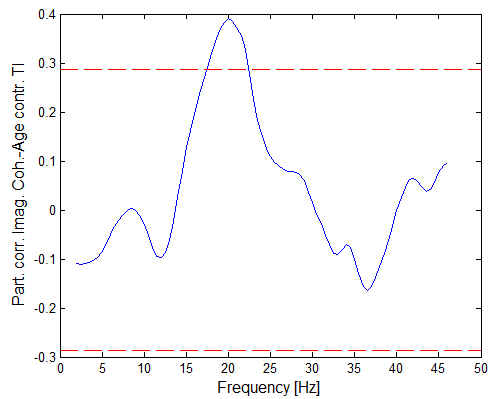

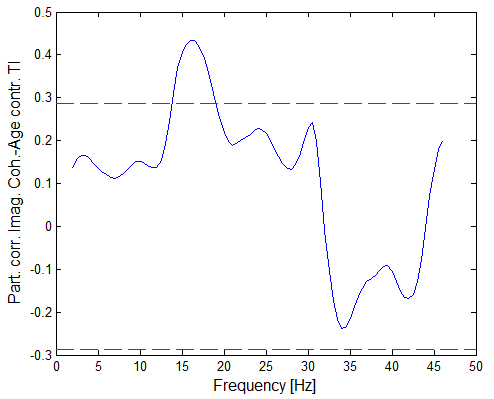


Cluster p-value: p = 0.11 p = 0.06

Between sensory and global component


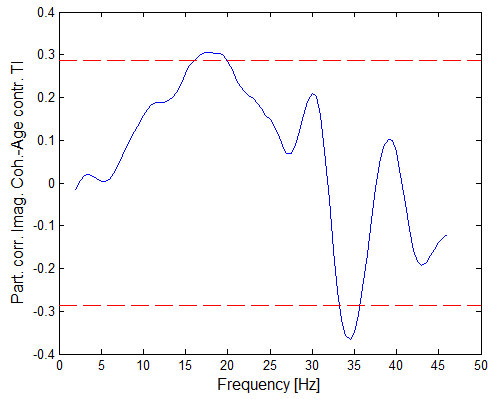


p-values: 0.24, 0.31

*Effective connectivity:*

Within sensory component Within global component


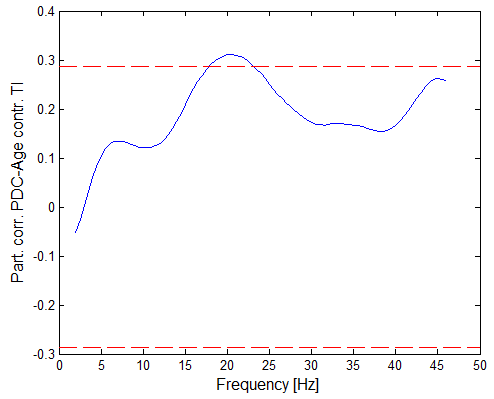

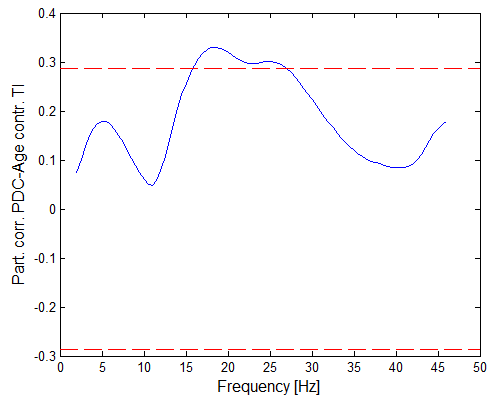


p-value: 0.12 p-value: 0.07

Outflow from ACs Inflow into ACs


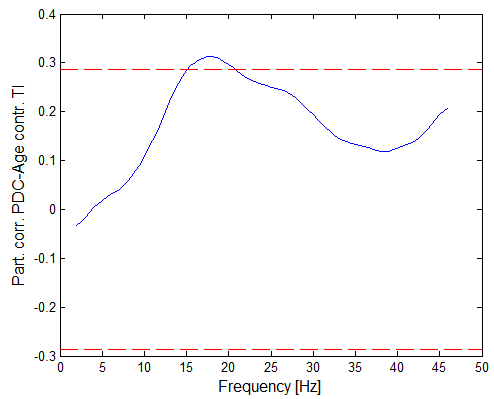

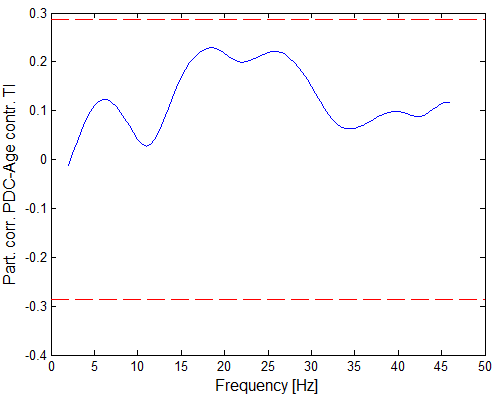


p-value: 0.12

***Conclusion:***

The above results show that there is no statistical evidence of an effect of Age on the primary outcome measures. The partial correlations remain small and exceed the single-comparison critical threshold only in narrow frequency intervals, if at all. All cluster p-values are above 0.05.

***References***

1. Weisz N, Müller S, Schlee W, Dohrmann K, Hartmann T, et al. (2007) The neural code of auditory phantom perception. Journal of Neuroscience 27: 1479-1484.

2. Anderson MJ, Robinson J (2001) Permutation tests for linear models. Australian & New Zealand Journal of Statistics 43: 75-88.

3. Freedman D, Lane D (1983) A nonstochastic interpretation of reported significance levels. J Bus Econom Statist 1: 292-298.
