# Supplementary material for: Source Space Estimation of Oscillatory Power and Brain Connectivity in Tinnitus
Source: PLoS One. 2015 Mar 23;10(3):e0120123. doi: 10.1371/journal.pone.0120123 (PMC4370720; doi:10.1371/journal.pone.0120123)
Supplement: S3 Appendix — (DOCX) [file pone.0120123.s006.docx]

**Appendix S3 - Confounding effects of hearing loss**

As there is a large difference in the pure-tone audiograms between the TI subjects and the controls, we have carried out analyses in order to assess the existence of confounding effects of hearing loss. The calculations were performed analogously to the ones for Age described in Appendix S2, i.e., ANCOVA- and cluster- based analyses for the primary outcome measures with subject group (controls vs TI) as fixed factor and Hearing Level as covariate. The Hearing Level covariate was computed as average over the measured levels between 0.25 and 4 kHz.

***Results:***

The diagrams below show the results of the ANCOVAs for the primary outcome measures with subject group (controls vs TI) as fixed factor and Hearing Level as covariate. We find that there is no significant effect of Hearing Level for both functional and effective connectivity. For the power spectra, however, the partial correlations between power and Hearing Level after controlling for TI are near or above 0.3 across wide ranges of frequencies for both sensory and global component, and there is a significant cluster of frequencies in the high beta and low gamma bands within the global component. This indicates that Hearing Level may have an effect on the power spectra. We have therefore reassessed the effect of tinnitus using the ANCOVA approach with Hearing Level as covariate. As shown in the final two diagrams, we still do not find a statistically effect of TI on spectral power. If anything, there is a slight indication of a reduction in power at low frequencies, contrary to the theoretical expectation.

*Spectra*

Within ACs Global component


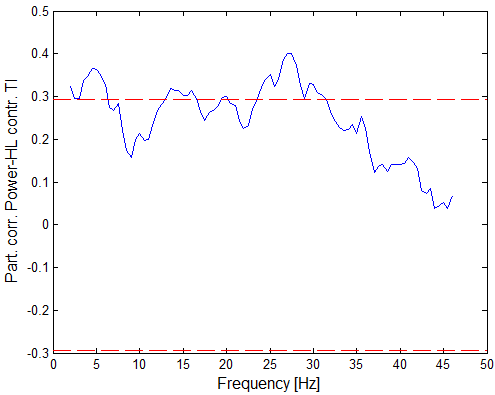

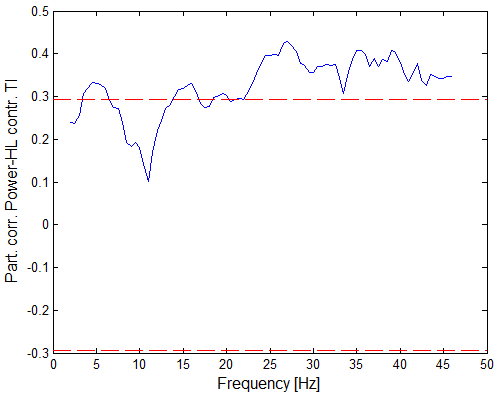


p-values = 0.147, 0.180, 0.290, 0.084 p-values = 0.148, 0.148, 0.175, 0.014

*Functional connectivity*

Within sensory component Within global component


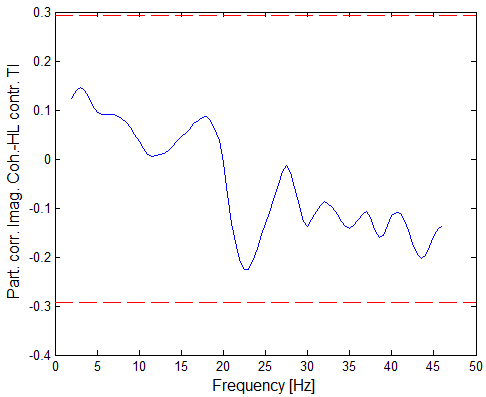

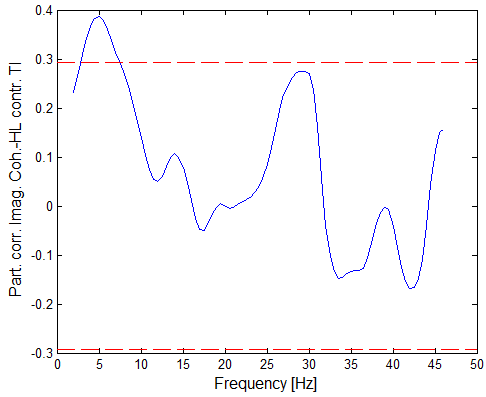


p = 0.148

Between sensory and global component


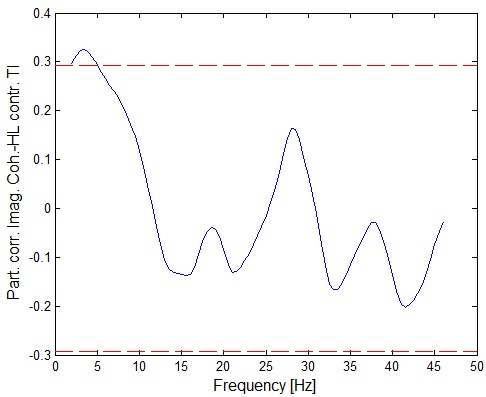


p = 0.266

*Effective connectivity:*

Within sensory component Within global component


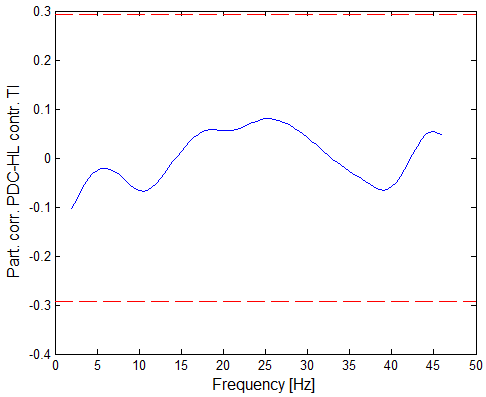

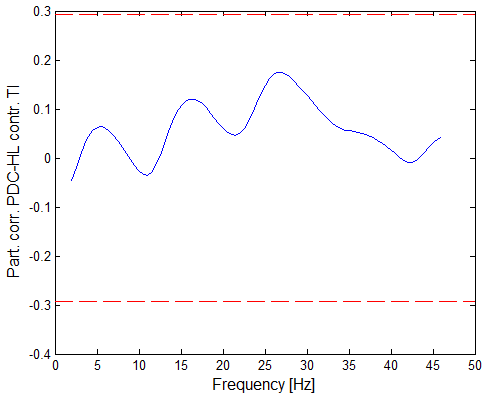


Outflow from ACs Inflow into ACs


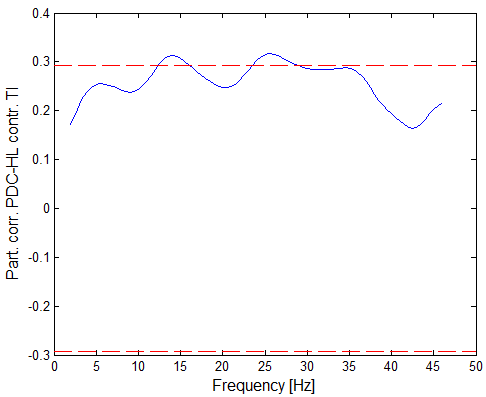

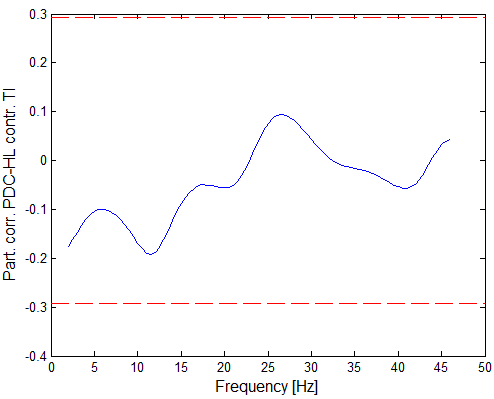


p-values = 0.127, 0.109

*Effect of TI: Comparing spectra after correcting for hearing loss*

Within sensory component Within global component


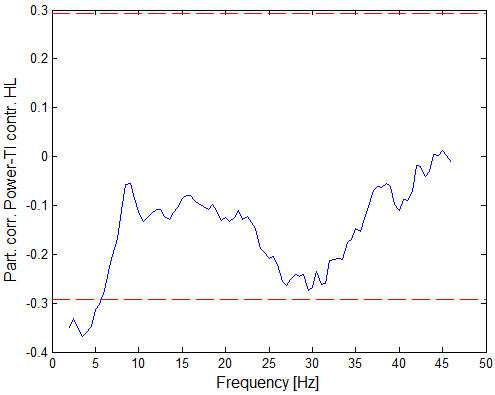

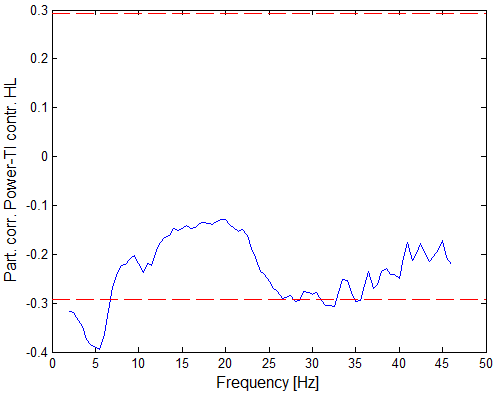


p = 0.149 p = 0.100, 0.228, 0.174, 0.228
