# Supplementary material for: Source Space Estimation of Oscillatory Power and Brain Connectivity in Tinnitus
Source: PLoS One. 2015 Mar 23;10(3):e0120123. doi: 10.1371/journal.pone.0120123 (PMC4370720; doi:10.1371/journal.pone.0120123)
Supplement: S4 Appendix — (DOCX) [file pone.0120123.s007.docx]

**Appendix S4 – Effects of noise contamination**

For many subjects, the sensor signals appear to be contaminated with additional narrow-band noise whose origin is unclear. This noise usually appears at narrow intervals near 21 and 34 Hz and occasionally also at other frequencies. It becomes visible in the spectra if the signals are Fourier-transformed over very long time windows in order to obtain the necessary frequency resolution. Further examination has indicated that the noise might be associated with specific sensors in the MEG helmet; however, this is not completely clear.

As it was feared that this noise might affect the analyses, it was eliminated by applying suitably defined narrow band-stop filters. It is clear that the analyses are not valid for frequencies near the filters. In order to assess whether filtering would also affect the results in a wider frequency region, the analyses were rerun using signals for which the additional noise has not been filtered out (note that a bandstop filter around 50 Hz was still applied to remove line noise which is not eliminated completely by the bandpass filter between 0.5 and 48 Hz). Every other processing step was performed in exactly the same way as in the analyses reported in the main manuscript. The comparison between the results with and without filter allows to assess the effect of the filtering.

***Results:***

Results show the comparisons for the various types of power spectra and connectivities considered in the primary analyses (blue/black– controls excluding/including noise [red/green for TI]), together with the observed t-statistic which are crucial for the cluster-based permutation analysis (solid/dashed curves – excluding/including noise).

*Power spectrum - Sensory component:*


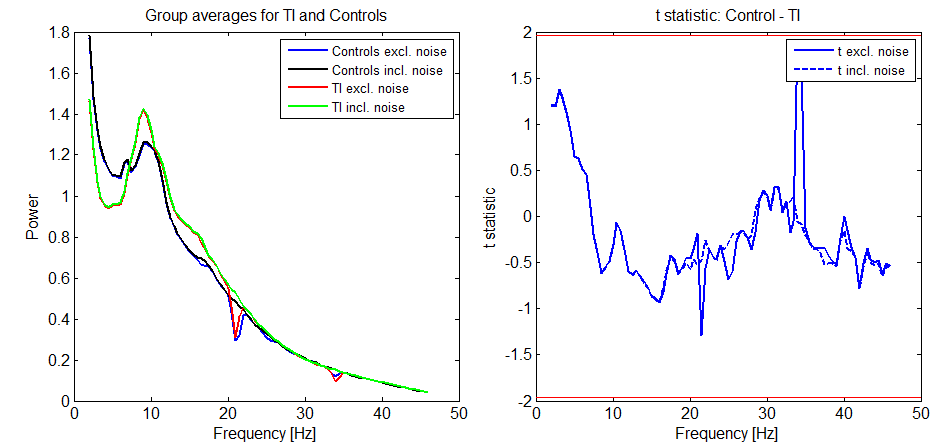


*Power spectrum – global component:*


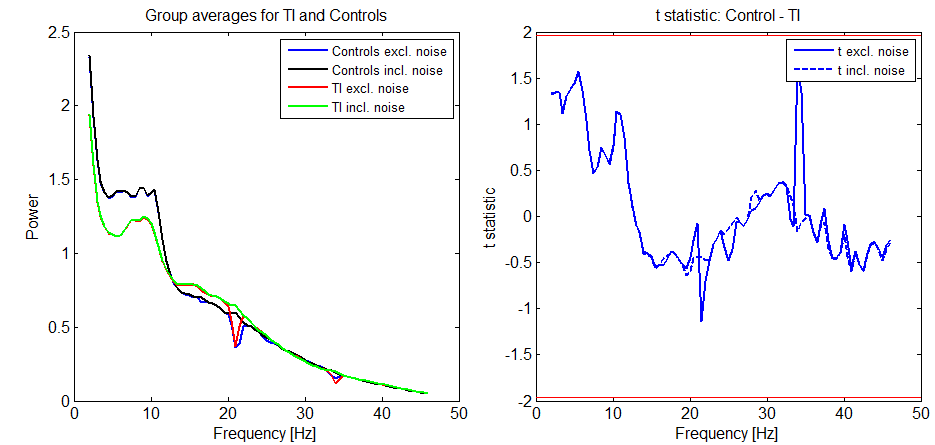


*Functional connectivity – within ACs:*


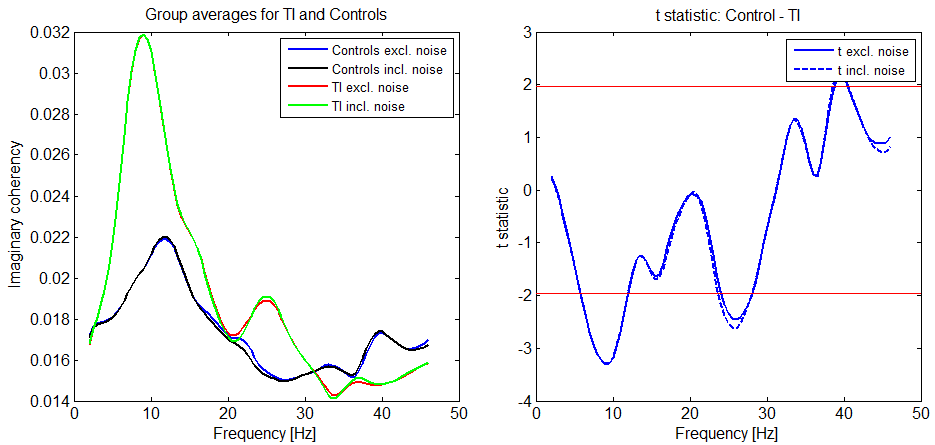


*Functional connectivity – within global components:*


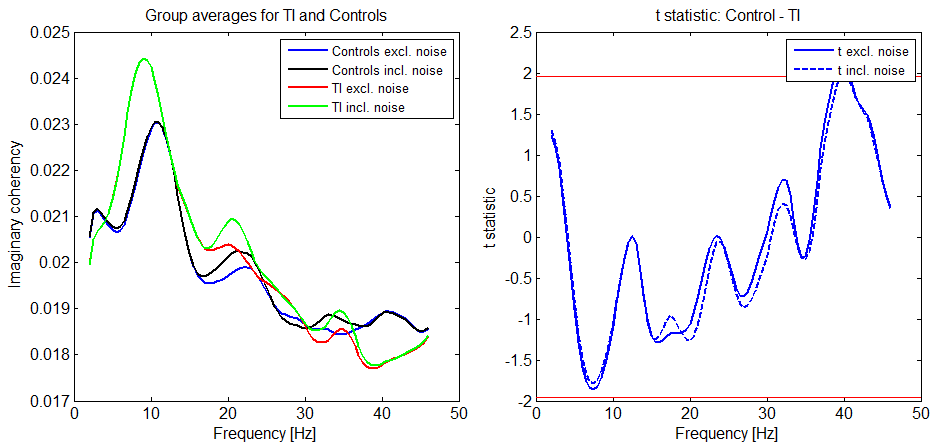


*Functional connectivity – between sensory and global component:*


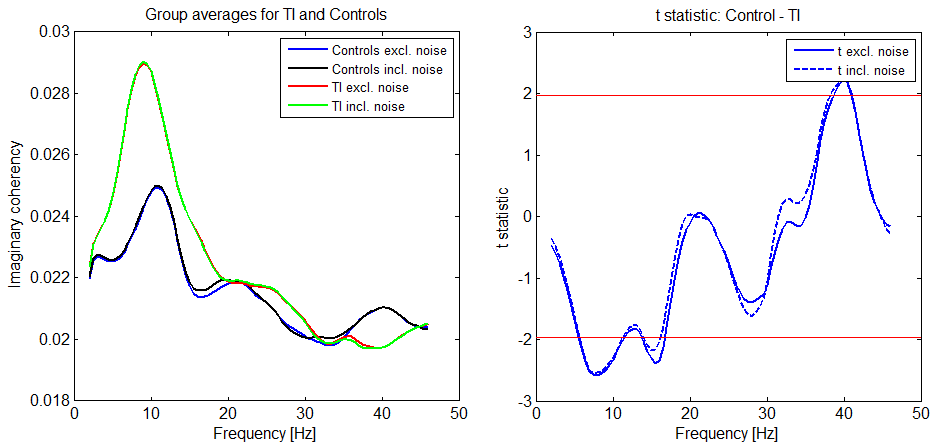


*PDC – within sensory component:*


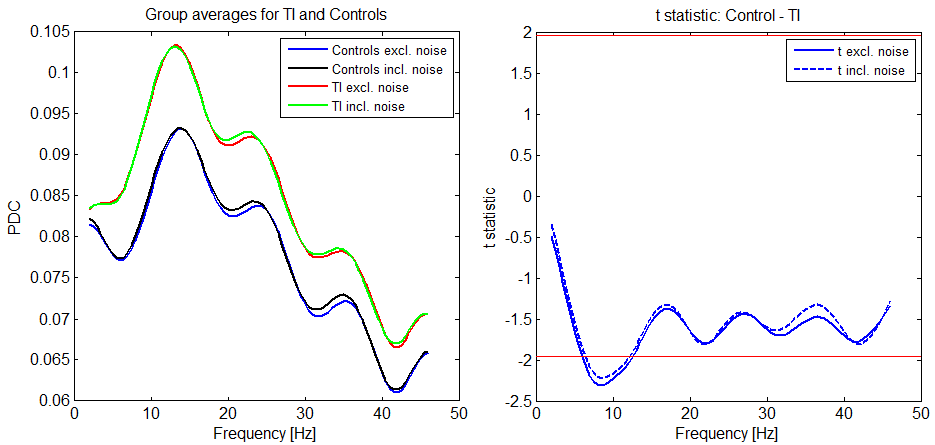


*PDC – within global component:*


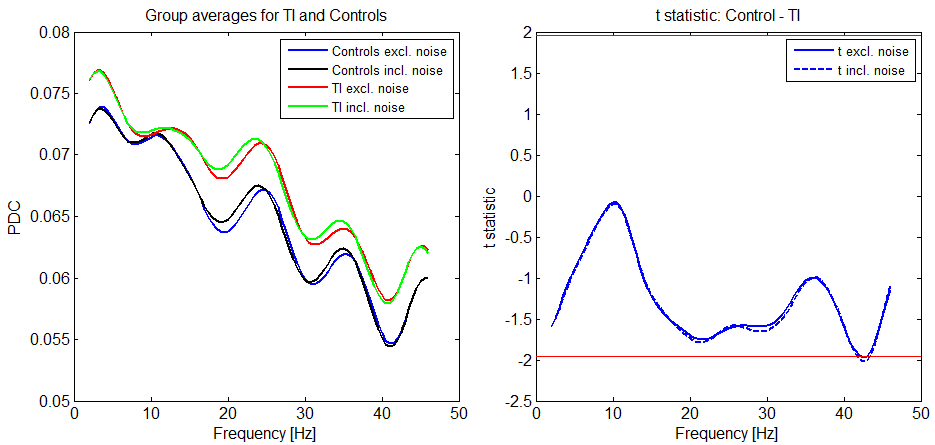


*PDC – outflow from sensory to global component:*


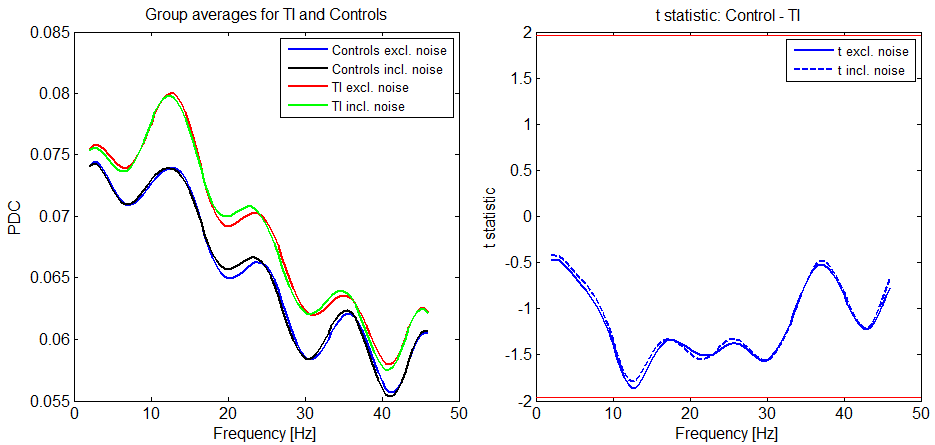


*PDC – global to sensory:*


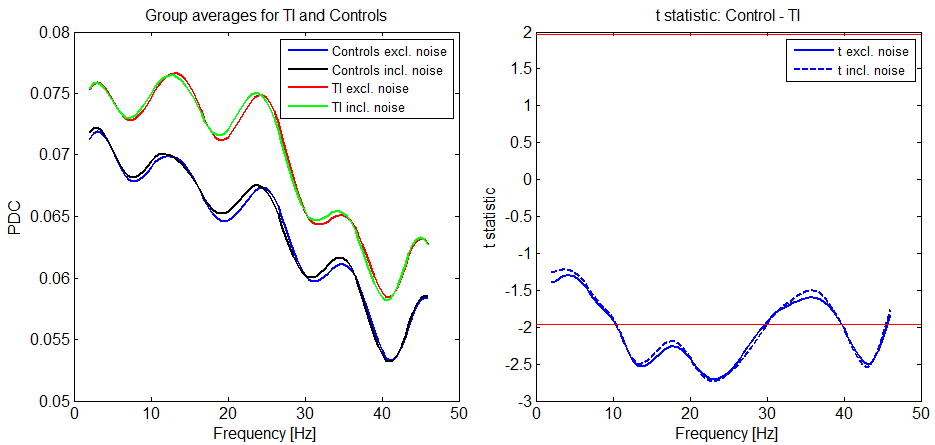


***Conclusion:***

Removal of the additional noise does not have an appreciable influence on the results of the primary analyses.

For the spectra, the effect of the removal remains strictly confined to the narrow frequency intervals around 21 Hz and 34 Hz where the noise is located. For the connectivities, the effects can indeed be more far-reaching in frequency for some subjects. However, effects are minimal in the delta, theta and alpha bands for the individual subjects, and after group averaging, the differences in the means and the t-statistics (which are crucial for detecting significant differences) are very small at all frequencies. It thus does not make any difference whether one uses the data with or without filtering.
