# Supplementary material for: Source Space Estimation of Oscillatory Power and Brain Connectivity in Tinnitus
Source: PLoS One. 2015 Mar 23;10(3):e0120123. doi: 10.1371/journal.pone.0120123 (PMC4370720; doi:10.1371/journal.pone.0120123)
Supplement: S5 Appendix — (DOCX) [file pone.0120123.s008.docx]

**Appendix S5 - Justification of the random-voxel-picking approach**

Several supplementary analyses were conducted in order to validate the random voxel-picking method used for computing the oscillatory spectra and connectivities.

*Spectra*. The spectra for the sensory and global components were recomputed using *all voxels* located in the regions of interest. More precisely, the calculation proceeded as follows:

1. Compute the power spectra for all voxels of the original 4x4x4mm^3^ grid that are located in the Brodmann areas pertaining to the regions of interest as listed in Table 2 of the main paper.
2. Compute an average spectrum for each BA by taking the mean over the spectra for all the voxels in this BA.
3. Using these BA spectra, spectra for the ROIs and the top-level components are computed by successive averaging as described in Sec. 2.3 of the main paper.

The current calculation thus differs from the approach of the paper only in that all voxels of a BA are used to calculate the BA spectrum rather than a random selection.

*Functional connectivity*. Two additional analyses were conducted to assess the sensitivity of our reported results to the method of voxel selection. (i) Following a suggestion of Hillebrand et al. [[1](#_ENREF_1)], we determined for each BA and frequency the voxel whose signal correlates most strongly with all other voxels in the BA. Functional connectivities between BAs are computed as connectivities between these voxels. (ii) For each BA, we selected 20 voxels that are spread out evenly across its constituent grid points. For each pair of BAs, the functional connectivity was determined as the average over the connectivities for all 20x20 pairs of voxels. Ideally, connectivities would be calculated by averaging over all pairs of voxels between BAs [[2](#_ENREF_2)]. However, such an approach is infeasible as computation times would be excessively long. With 20 voxels per BA, computations are still practicable and it seems plausible that the voxels capture the variations across the BA well (there are between 2 and 244 voxels per BA). Compared to the original method, this approach should be more accurate as it is based on 20 voxels selected to be spread out across the BA rather than 10 randomly drawn ones, and it uses 20x20=400 connectivities rather than 10 (each random voxel in one BA is paired with one from the other BA). However, computation times are increased by a factor 10-15.

Further details of these new approaches are as follows.

(i) To determine the voxel with maximum within-BA correlation for a given BA and frequency, the coherence matrix (i.e., the absolute value of the normalized cross-spectrum) for all voxels in the BA at that frequency was computed. For each voxel, the coherences with the other voxels were summed up and the voxel with maximum summed coherence was selected as representative of the BA. Connectivities between BAs were then computed as connectivities between the representative voxels.

However, since the representative voxels often change from one frequency to the next, there can be appreciable fluctuation in the plots of connectivity versus frequency. Some degree of smoothing was therefore introduced in the following way. At each frequency, the BA-BA connectivity is determined as the weighted average of the connectivity for the representative voxel pairs at that frequency as well as for the pairs at 4 adjacent frequencies below and above the target frequency (frequencies are in intervals of 0.5Hz). This is motivated by the idea that a voxel representative at a certain frequency should remain to be representative at nearby frequencies (even if some other voxels are even “more representative” at these frequencies). More precisely, the calculation proceeds as follows.


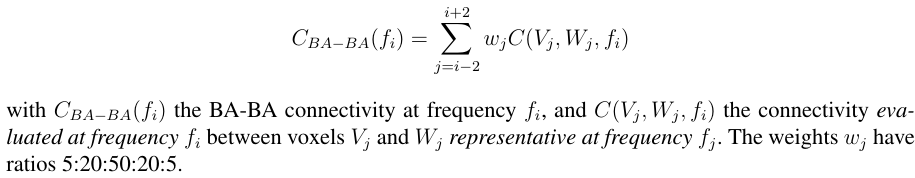


Note that the voxels *V_j_* (and *W_j_*) need not be all different from each other.

(ii) For a given BA, the 20 voxels are selected as follows. A principal components analysis is conducted on the positions of all voxels in the BAs, and the voxels are ranked according their coordinate on the first principal component (which gives the direction of largest spread within the cloud of voxels). The 20 voxels are then selected such that they are evenly spread across the rank order (e.g., every fifth if there are 100 voxels altogether in the BA). Plots show that with this approach the selected voxels tend to be evenly spread out over the spatial extent of the BA. If a BA contains 20 voxels or less, all voxels are used.

After determining the BA-BA connectivities, the subsequent calculations proceed as described in Sec. 2.3 of the main paper.

***Results***

*Comparison of alternative methods to random-voxel results*

Diagrams show the comparisons for the various types of power spectra and functional connectivities considered in the primary analyses (blue/black – random-voxel/modified results for controls [red/green for TI]), together with the observed t-statistic which are crucial for the cluster-based permutation analysis (solid / dashed curves – random-voxel / modified results).

*Spectra using* ***all voxels*** *– sensory component*


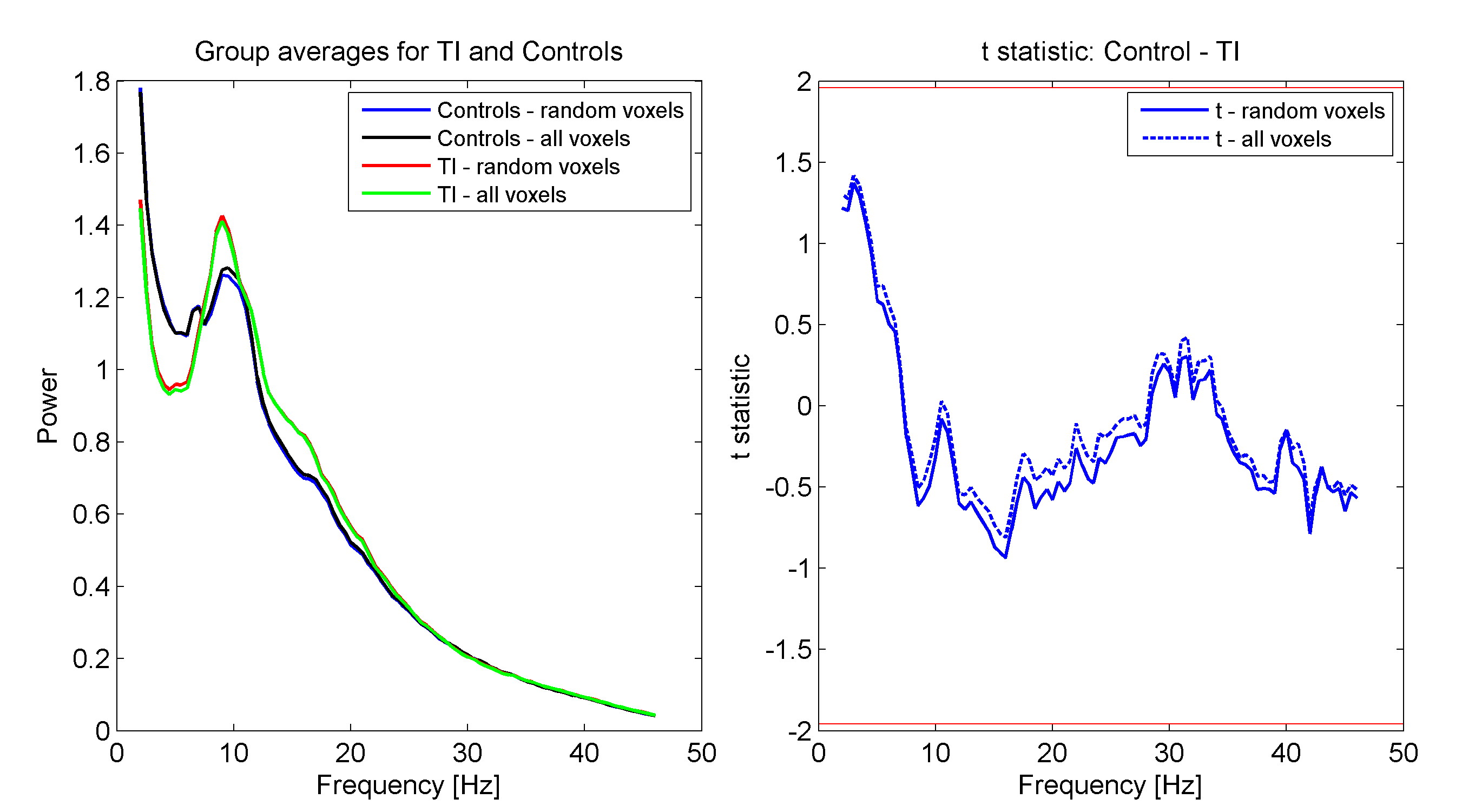


*Spectra using* ***all voxels*** *– global component*


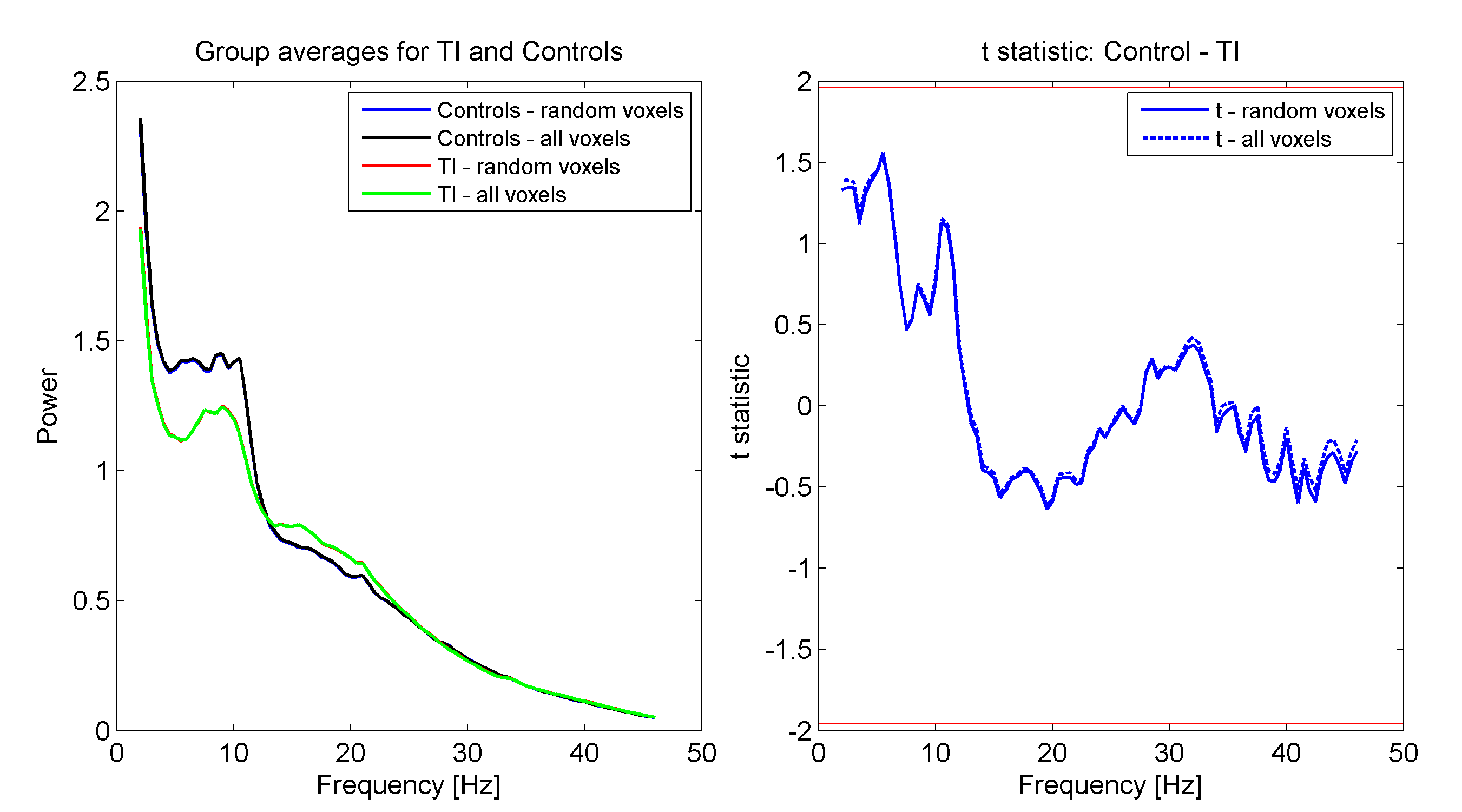


*Functional connectivity using* ***20 distributed voxels*** *– within sensory component*


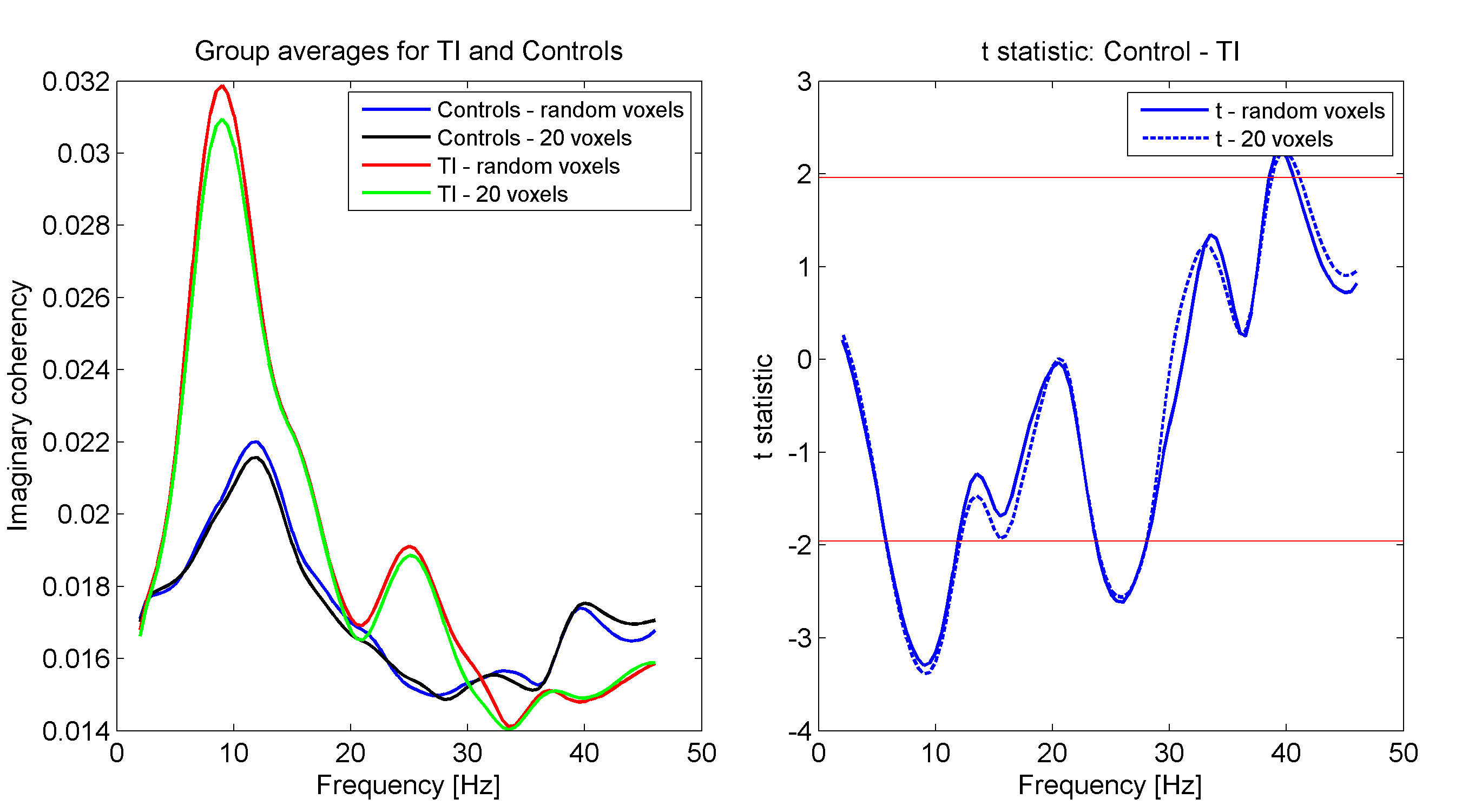


*Functional connectivity using* ***20 distributed voxels*** *– within global component*


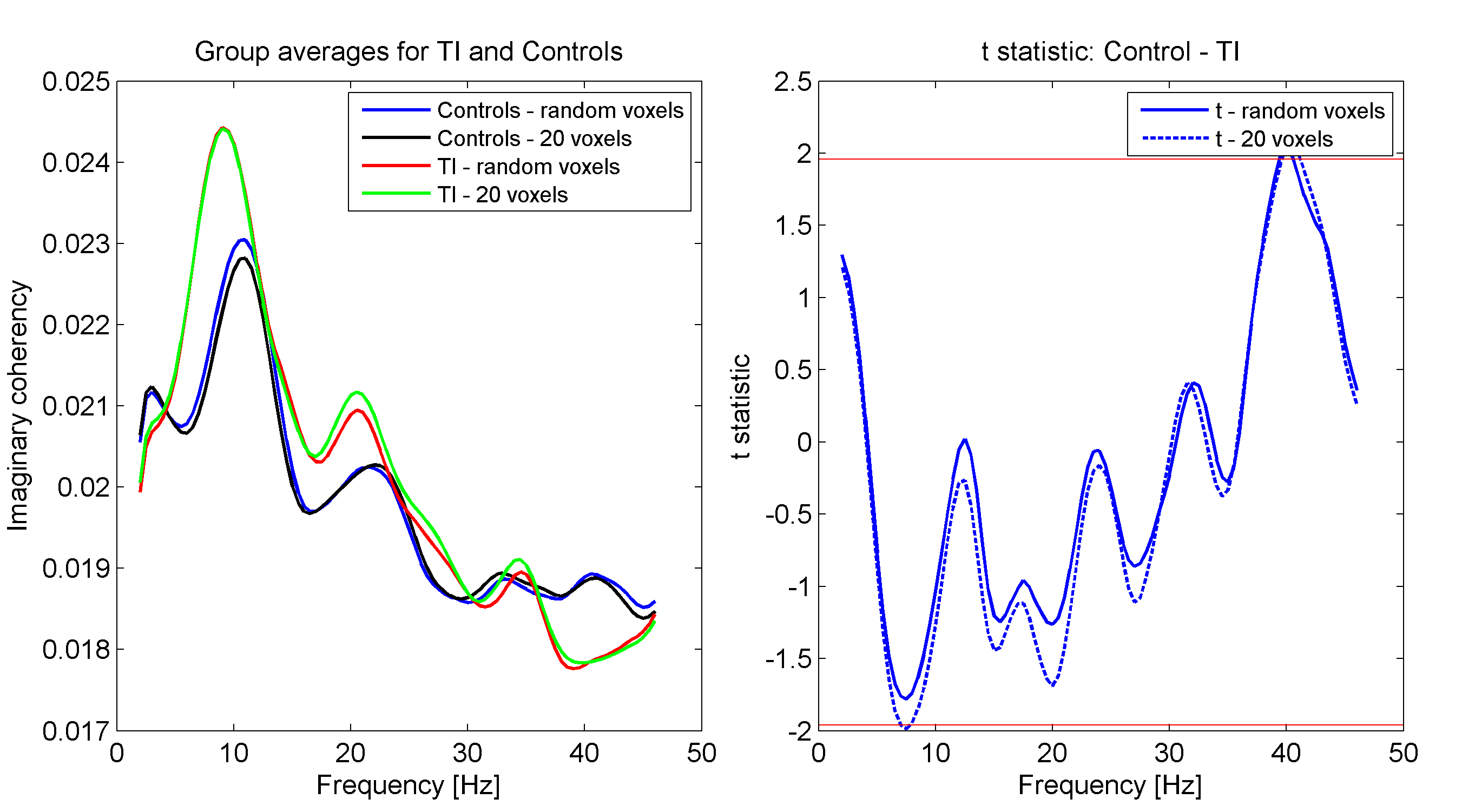


*Functional connectivity using* ***20 distributed voxels*** *– between sensory and global component*


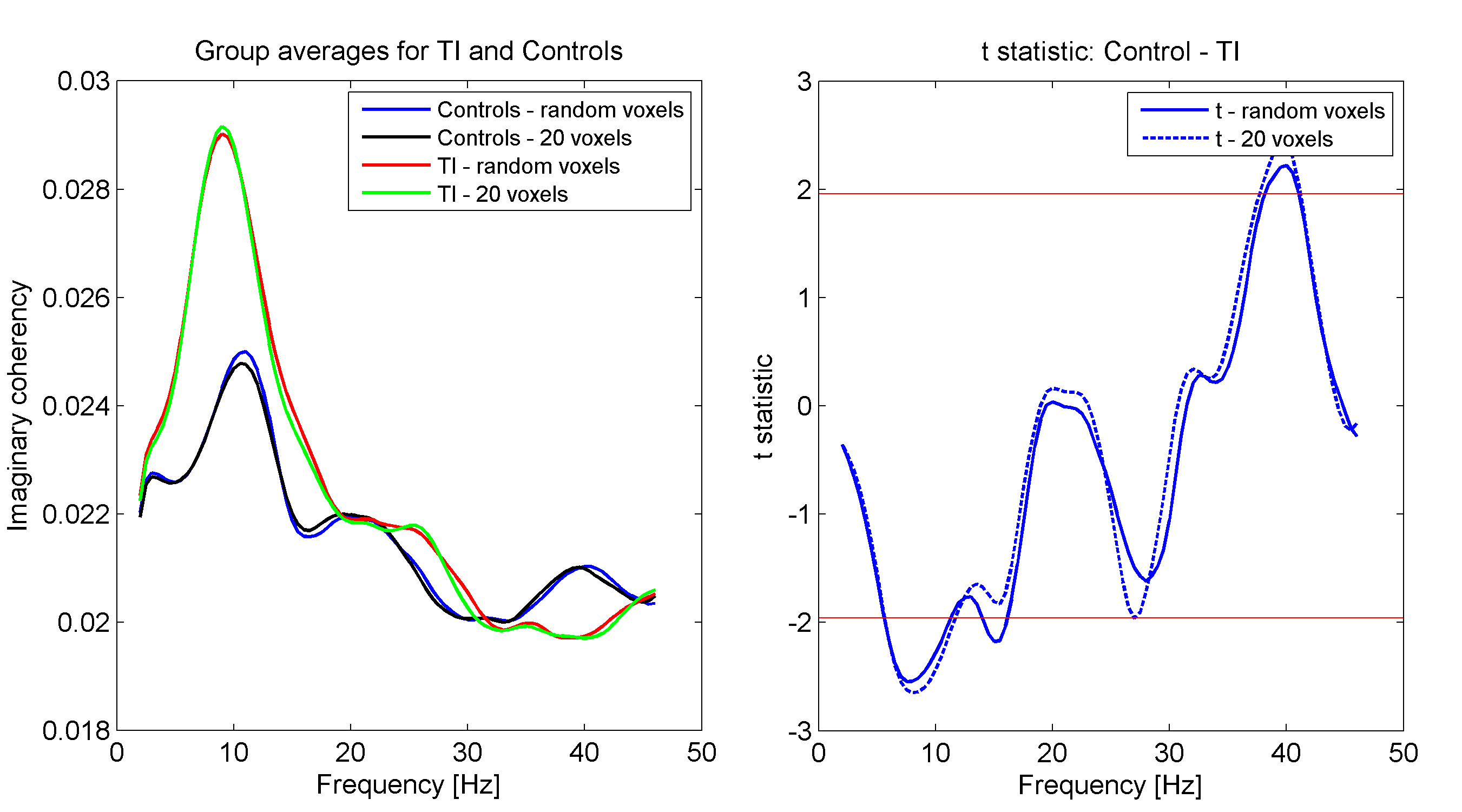


*Functional connectivity with* ***maximum correlation voxels*** *– within sensory component*


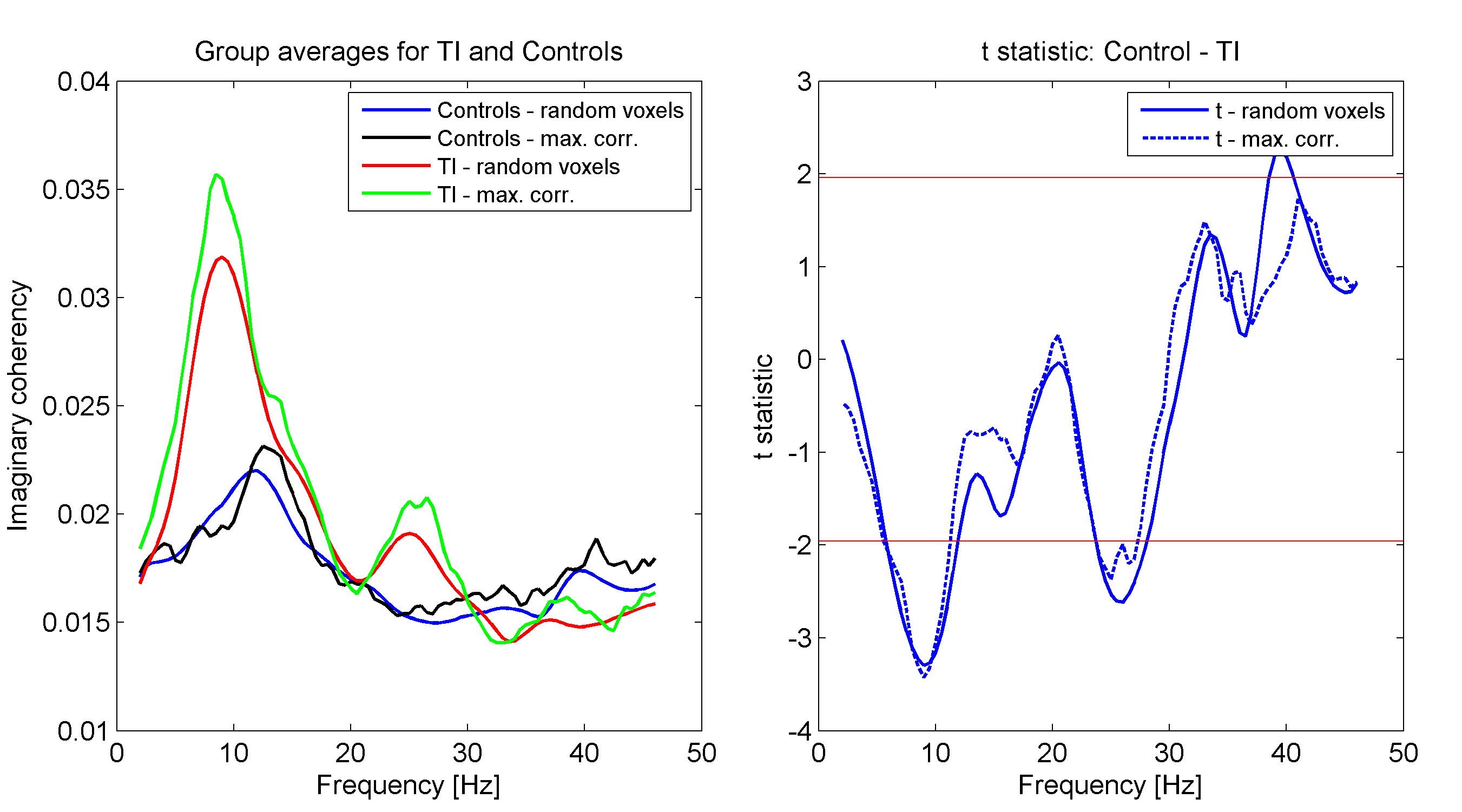


*Functional connectivity with* ***maximum correlation voxels*** *– within global component*


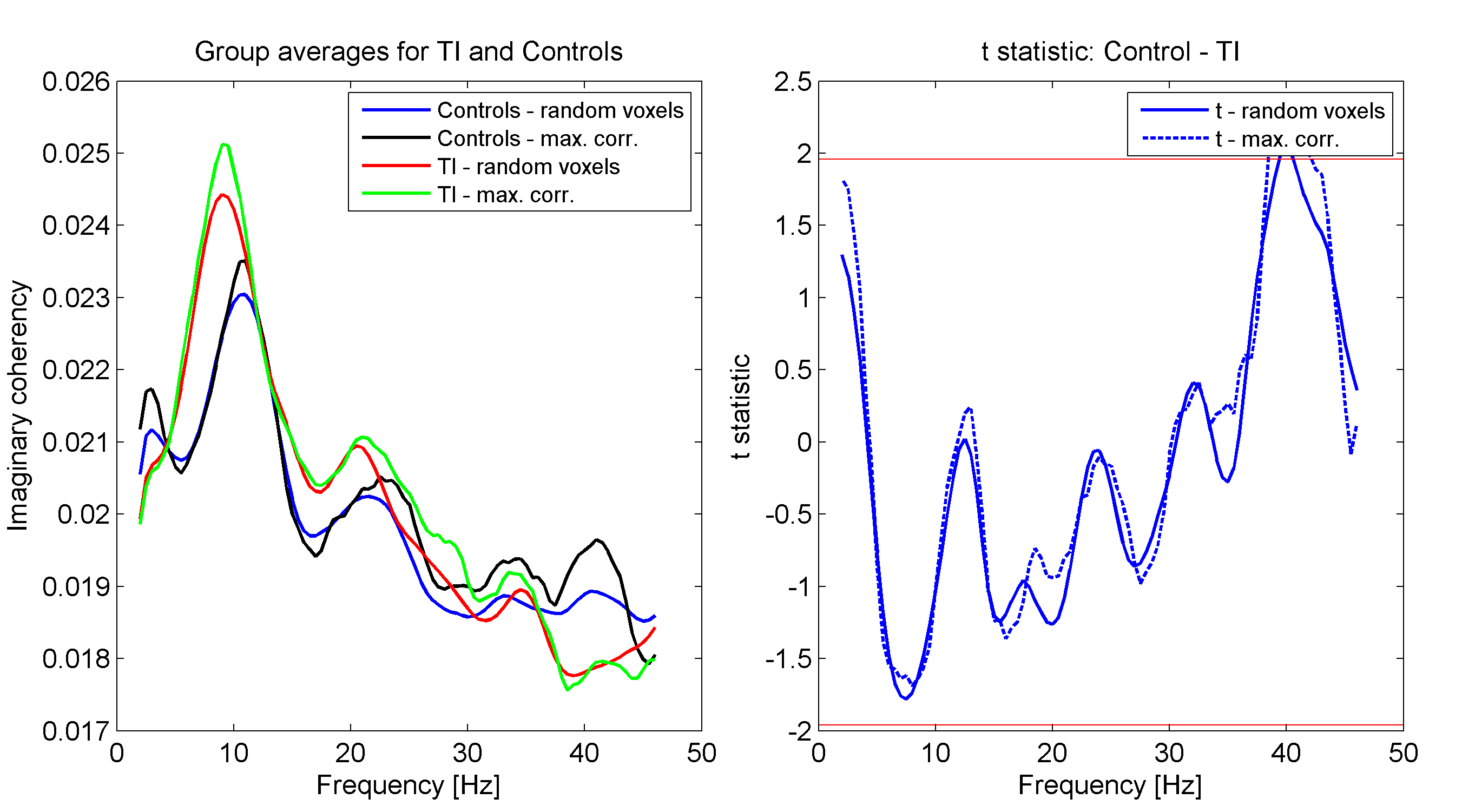


*Functional connectivity with* ***maximum correlation voxels*** *– between sensory and global component*


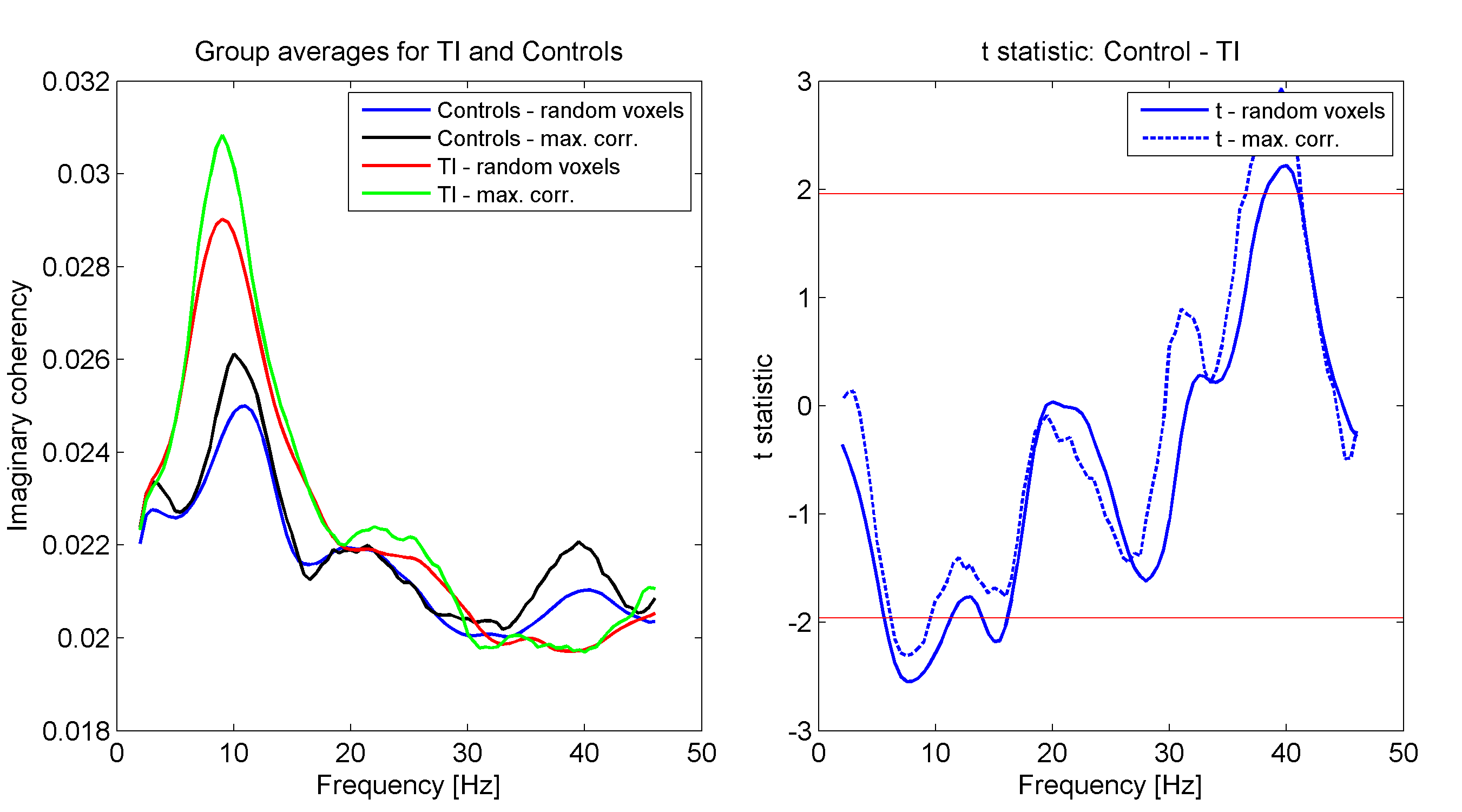


*Voxel-selection example – 20 voxels spread out across BA:*


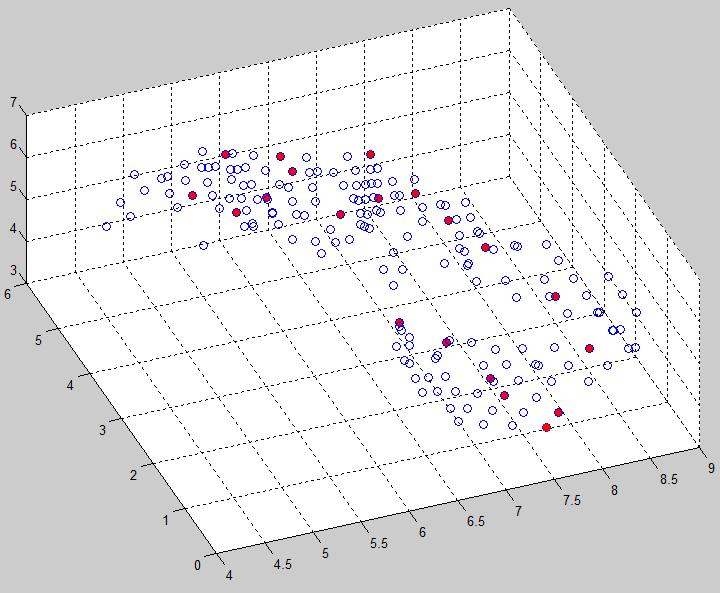


***Conclusions:***

The reported spectra are very well reproduced by the new calculation using all voxels. The same is observed for the connectivities with the calculation using the 20-voxels set. For the approach using the maximum-correlation voxels, the differences are slightly larger. However, we still observe very good agreement and any deviations are quantitative rather than qualitative. All the main features in the diagrams are reproduced and the t-statistics which eventually determine the statistically significant differences are very similar.

These results validate our random voxel-picking method and show that our results are reliable and not unduly affected by artefacts of voxel selection.

Note that at this stage, we have not performed any further analysis for PDC. Such investigations would be more intricate as, in order to produce results comparable to the original analysis, each BA has to be represented by a single voxel across all frequencies and one has to select voxels for all BAs simultaneously. The above methods for functional connectivity thus cannot be transferred directly. Nevertheless, based on the positive results for the spectra and functional connectivities, we are confident that our approach is also valid for PDC.

***References***

1. Hillebrand A, Barnes GR, Bosboom JL, Berendse HW, Stam CJ (2012) Frequency-dependent functional connectivity within resting-state networks: An atlas-based MEG beamformer solution. Neuroimage 59: 3909-3921.

2. Palva JM, Monto S, Kulashekhar S, Palva S (2010) Neuronal synchrony reveals working memory networks and predicts individual memory capacity. Proceedings of the National Academy of Sciences of the United States of America 107: 7580-7585.
